# Surface faulting earthquake clustering controlled by fault and shear-zone interactions

**DOI:** 10.1038/s41467-022-34821-5

**Published:** 2022-11-21

**Authors:** Zoë K. Mildon, Gerald P. Roberts, Joanna P. Faure Walker, Joakim Beck, Ioannis Papanikolaou, Alessandro M. Michetti, Shinji Toda, Francesco Iezzi, Lucy Campbell, Kenneth J. W. McCaffrey, Richard Shanks, Claudia Sgambato, Jennifer Robertson, Marco Meschis, Eutizio Vittori

**Affiliations:** 1 School of Geography, Earth and Environmental Sciences, University of Plymouth, Drake Circus, Plymouth, PL4 8AA UK; 2 Department of Earth and Planetary Sciences, Birkbeck, University of London, London, WC1E 7HX UK; 3 IRDR, University College London, Gower Street, London, WC1E 6BT UK; 4 Computer, Electrical and Mathematical Sciences and Engineering, 4700 King Abdullah University of Science and Technology (KAUST), 23955-6900 Thuwal, Kingdom of Saudi Arabia; 5 Laboratory of Mineralogy and Geology, Department of Natural Resources Development & Agricultural Engineering, Agricultural University of Athens, 11855 Athens, Greece; 6 Dipartimento di Scienza ed Alta Tecnologia, Università degli Studi dell’Insubria, Como, Italy; 7 Istituto Nazionale di Geofisica e Vulcanologia, Sezione di Napoli Osservatorio Vesuviano, Via Diocleziano 328, 80124 Naples, Italy; 8 International Research Institute of Disaster Science, Tohoku University, Sendai, Japan; 9 DiSTAR—Dipartimento di Scienze della Terra, dell’Ambiente e delle Risorse, University of Naples “Federico II”, Naples, Italy; 10 School of Environmental Sciences, University of Hull, Hull, HU6 7RX UK; 11 Department of Earth Sciences, University of Durham, Durham, DH1 3LE UK; 12 Scottish Universities Environmental Research Centre, Glasgow, G75 0QF UK; 13 Istituto Nazionale di Geofisica e Vulcanologia, Sezione di Palermo, Via Ugo La Malfa, 153, 90146 Palermo, Italy; 14 CNR, Institute of Geosciences and Earth Resources, Via La Pira 4, 50121 Florence, Italy

**Keywords:** Tectonics, Natural hazards, Structural geology

## Abstract

Surface faulting earthquakes are known to cluster in time from historical and palaeoseismic studies, but the mechanism(s) responsible for clustering, such as fault interaction, strain-storage, and evolving dynamic topography, are poorly quantified, and hence not well understood. We present a quantified replication of observed earthquake clustering in central Italy. Six active normal faults are studied using ^36^Cl cosmogenic dating, revealing out-of-phase periods of high or low surface slip-rate on neighboring structures that we interpret as earthquake clusters and anticlusters. Our calculations link stress transfer caused by slip averaged over clusters and anti-clusters on coupled fault/shear-zone structures to viscous flow laws. We show that (1) differential stress fluctuates during fault/shear-zone interactions, and (2) these fluctuations are of sufficient magnitude to produce changes in strain-rate on viscous shear zones that explain slip-rate changes on their overlying brittle faults. These results suggest that fault/shear-zone interactions are a plausible explanation for clustering, opening the path towards process-led seismic hazard assessments.

## Abstract (web summary)

The mechanisms responsible for clustering of surface fault earthquakes are often unclear. Here the authors find that differential stress fluctuates during fault/shear-zone interactions which can produce changes in strain-rate and slip-rate changes leading to earthquake clustering.

## Introduction

It has long been known that earthquake recurrence is not strictly periodic, with evidence for temporal earthquake clusters and elevated slip rates lasting hundreds to thousands of years and containing several large-magnitude (M_w_ > 6) earthquakes on single faults, separated by times of relative fault quiescence^1,2^. Currently, we lack understanding of what controls such aperiodicity. This confounds our attempts to understand uncertainties and time-dependencies of seismic hazard, because the greater the uncertainty in aperiodicity, the greater the uncertainty in recurrence intervals, a vital input for time-dependent probabilistic seismic hazard assessment^3^.

The processes that produce slip-rate variations associated with the temporal clustering of surface faulting earthquakes are debated^2^, but include (1) fault interaction, (2) strain-storage in the crust and (3) evolving dynamic topography. Fault interaction occurs where slip on a fault deforms the surrounding volumes of rock, modifying stresses that alter the timing of slip on other structures in that volume^2,4,5^. Strain may be stored in the crust due to deformation, or microstructural evolution and/or fluid infiltration (rheological changes), both within the brittle fault zones and within their downward continuations in the viscous lower crust known as shear zones^2,6,7^. There may also be storage of residual elastic strain because strain release during individual earthquakes lags behind the rate of elastic strain accumulation during the preceding interseismic period^2,8^. Additionally, where dip-slip motion occurs across combined fault/shear-zone structures, this builds topography, and this in turn alters the stresses acting on fault/shear-zones and alter the potential for faulting and/or viscous slip and the timing of deformation pulses^9^. Although we have this understanding, we lack quantified examples where numerical models of the above processes replicate, and hence are calibrated by, measurements of earthquake clustering. Therefore, the relative contribution of the three processes listed above to earthquake clustering is unclear, which is a challenge to developing a process-led approach to seismic hazard analysis that includes clustering^2,3,10^.

The hypothesis we investigate is whether the changes in differential stress (defined as the difference between the largest (*σ*_1_) and smallest (*σ*_3_) principal stresses, *σ*_1_*–σ*_3_) produced by fault/shear-zone interactions are of sufficient magnitude to drive changes in strain-rate in viscous shear zones, that in turn drive periods of rapid or slowed slip on overlying brittle faults during clustering/anti-clustering. This hypothesis arises because we note that the middle crust (~15–24 km) is weaker than the upper crust so the former undergoes continuous viscous creep in shear zones that drives periodic brittle slip on overlying faults^11,12^. Slip on the combined fault/shear-zone structures will produce changes in differential stress on neighboring fault/shear-zones during their interaction, and differential stress is related to strain-rate in the viscous material^13^ by $\dot{\varepsilon }$ ∝ *σ*^*n*^, where $\dot{\varepsilon }$ is strain-rate, and *σ* is differential stress raised to the power *n*. Thus, changes in differential stress will produce changes in viscous strain-rate, but it is unclear whether the magnitudes of these changes are sufficient to drive the changes in slip-rate that occur over the time periods of hundreds to thousands of years associated with surface faulting earthquake clusters and anti-clusters.

**Fig. 1:**
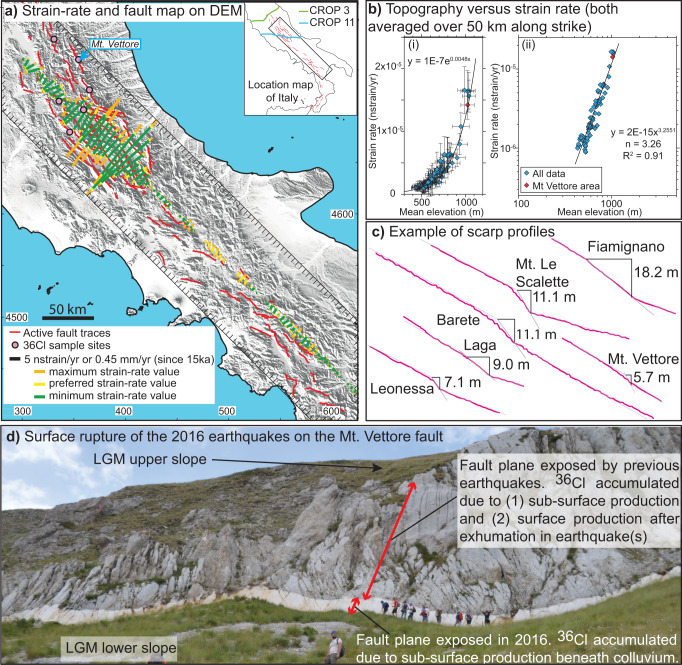
Current knowledge of fault and shear zone interaction in the central Apennines. **a** Map showing the spatial variation in principal horizontal strain rate (maximum, preferred and minimum strain-rate values shown) calculated on a 5 × 90 km grid (delineated by black lines and tick marks) traversing the Italian Apennines (topography from SRTM DEM), derived from the directions and magnitudes of faulted-offsets since 15 ± 3 ka of landforms dating from the demise of the Last Glacial Maximum, modified and updated from ref. 20 The locations of ^36^Cl sample site and deep seismic reflection datasets mentioned in the text are indicated in the inset map. **b** Topography against strain-rate from **a**, showing a power law correlation (ii) with an exponent of ~3.26 between datasets, updated from ref. 18. The value of this power law relationship exponent implies that the brittle faults are underlain and driven by viscous shear zones. The error bars in (i) are 95% confidence intervals of the mean elevations (assuming a normal distribution) and error in strain-rate propagated from field measurements (see refs. 20,35 for more detail). **c** Topographic profiles across active fault scarps used in this study. **d** Surface ruptures of the 2016 earthquakes on the Mt. Vettore fault scarp showing how slip on the brittle faults generates surface offsets whose timing and magnitude can be constrained via ^36^Cl analyses.

**Fig. 2:**
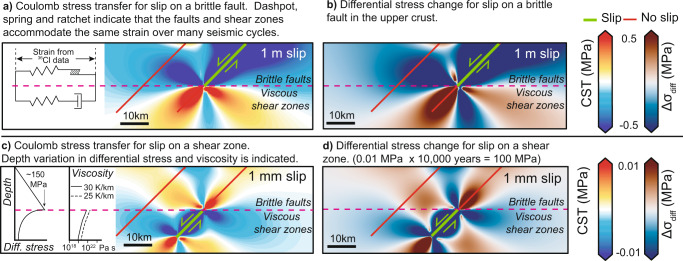
Model set-up used to examine stress changes produced by slip in normal faulting earthquakes and by slip on underlying shear zones. **a** shows Coulomb Stress Transfer (CST) resulting from a normal faulting earthquake. Inset shows the brittle-frictional-viscous components in the upper and middle crust. **b** shows the differential stress changes resulting from a normal faulting earthquake. **c** shows CST resulting from slip on a viscous shear zone. Insets show typical values for differential stress^19^ and viscosity^24^ (for two isotherms) with depth. Note that the part of the shear zone that is most resistant to deformation (i.e., rate-limiting) will be the shallowest part due to the highest viscosity. **d** shows the differential stress resulting from slip on a viscous shear zone. Both earthquakes and shear zone slip transfer negative differential stress (a reduction in stress) onto the neighboring shear zone, so a change in strain-rate on the receiver shear zone is implied.

**Fig. 3:**
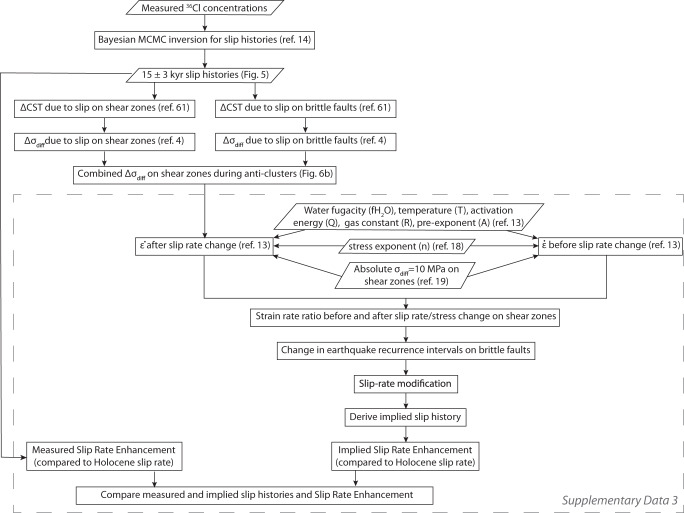
Model workflow to link the surface findings (^36^Cl-derived slip histories) with modeling stress changes and calculating the resulting changes in strain-rates and slip-rates. Parallelograms represent inputs/outputs and rectangles represent processes in our modelling workflow. The steps contained within the grey dashed line are contained within Supplementary Dataset 3.

**Fig. 4:**
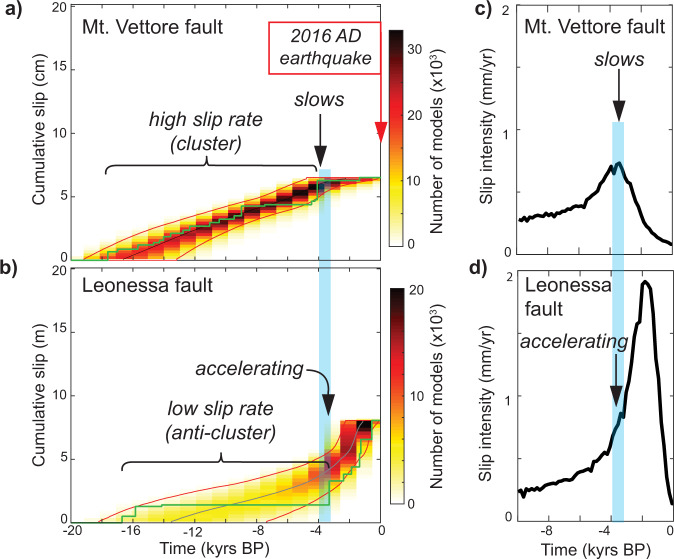
Slip histories for the Mt. Vettore and Leonessa faults that are located across strike from each other. **a**, **b** show the best least squares slip histories with stepped green lines, and the color scale indicates the top 100,000 (after model burn-in) least squares slip histories for each fault. These represent the best fits to the data without penalization of results by priors used in the modeling during the operation of the Markov chains. We also show the 90% confidence bounds (red lines) derived from the posterior distributions produced by the Bayesian modeling. **c**, **d** show the mean slip intensity results for the same model runs as **a** and **b**, but here choices of runs are penalized by uncertainties indicated by priors placed on the modeling, where slip intensity is size of slip events multiplied by their frequency divided by bin size for all model runs in the posterior distribution. Bin size is defined as the optimal bin size for the distributions^14^. Both least squares and highest likelihood results reveal out-of-phase slip on these two faults, with high slip-rate on one accompanied by lower slip-rate on the other, indicating interaction between these two structures to maintain the regional strain-rate indicated in Fig. 1.

**Fig. 5:**
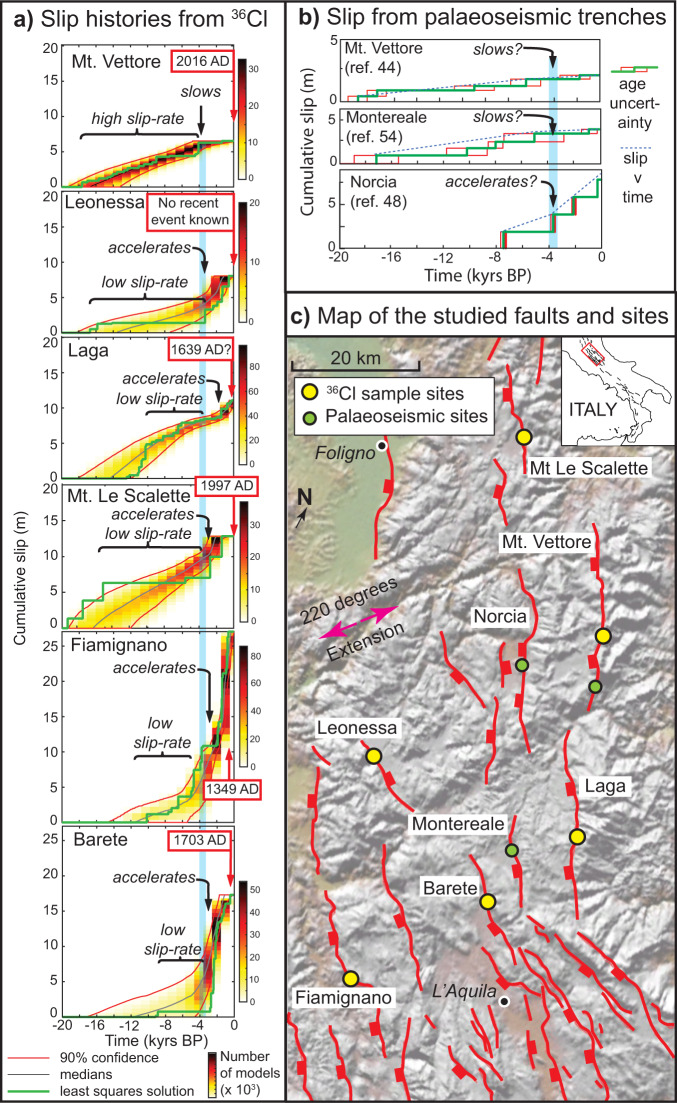
Slip histories for individual faults in the study region. **a** Slip histories derived from in situ ^36^Cl cosmogenic exposure data for the six faults studied. At ~3.5 kyrs BP (~3.5 ka), the least squares slip histories, the ensembles of least squares slip histories, and the 90% confidence bands exhibit convex upward shapes for the Mt. Vettore fault, and convex downward shapes for all the other faults. Concavity and convexity indicate that slip-rates change for all the faults at ~3.5 kyrs B.P.; the Mt. Vettore fault slows in activity and has a period of quiescence whilst all the other faults accelerate. Red boxes give the date of the last known earthquake on the faults studied. **b** Slip histories from some nearby faults from published paleoseismic trenching that broadly agree with our cosmogenic data. **c** Map showing the locations of the faults studied, ^36^Cl sample sites and paleoseismic trenches (topography from SRTM DEM).

In this work, we present our findings concerning slip-rate changes on brittle faults in central Italy (see location in Fig. 1a), and attempt to replicate the findings through modeling (Figs. 2 and 3). The slip-rates and slip-rate changes are derived from in situ ^36^Cl cosmogenic exposure dating of bedrock fault scarps (Figs. 4 and 5)^14^. The data confirm the slip-rates and strain-rates averaged over 15 ± 3 ka in Fig. 1, but reveal periods of rapid slip on some faults, with up to 15 m of slip in as little as 3500 years, that are contemporaneous with periods of low or no slip on neighboring faults across strike. The faults are relatively short, 20–40 km in length, and scaling between fault length and coseismic offsets suggests they should only be able to experience slip of ~1–2 m in a single earthquake^15^, so we interpret the periods of rapid slip as temporal earthquake clusters, and periods of low or no slip as anticlusters (following ref. 2). The finding that periods of rapid slip do not occur synchronously on all faults rules out a regional explanation for the rapid exposure of the fault planes^2,16^. Instead, periods of rapid slip are restricted to a sub-set of the faults, and these periods are contemporaneous with periods of low or zero slip on other faults (e.g., Fig. 4). Out-of-phase behavior observed on neighboring faults hints at interaction between these structures over millennial timescales^6,17^. Firstly, we show that the slip-rate changes must be accompanied by strain-rate changes on underlying shear zones, otherwise implausibly large stresses would build up on faults during anti-clusters lasting many millennia. Secondly, we present the results of modeling that links interaction between neighboring fault/shear-zone structures, strain-rate changes and slip-rate changes that produce earthquake clustering.

## Results

### Background to the modeling approach

An important insight into the potential cause(s) of clustering comes from recent work^18^, consistent with a classic idea^11,12^, that slip on brittle faults in the upper crust is driven by the slip on underlying viscous shear zones in the middle crust. On timescales longer than that for a single coseismic slip event, the upper crust is relatively strong compared to the middle crust because friction increases with depth before viscous deformation initiates due to increasing temperature with depth (Fig. 2c). For example, for the specific case of the Whipple extensional detachment in eastern California and Arizona, the upper crust has been shown to support differential stresses increasing from zero at the surface to ~100–150 MPa or more at its base at ~10–12 km, with values decreasing to as low as ~10 MPa over the ~12–20 km depth range where viscous flow initiates^19^. The change in crustal strength implies that slip on viscous shear zones occurs as creep during the build-up stresses that lead to earthquake rupture. In other words, the viscous slip drives the slip on the overlying brittle faults. Recent work^18^ confirms this relationship and the link to dynamic topography, because it revealed a correlation between strain-rates derived from measurements of slip-rates on surface fault scarps^20^ and topographic elevation in the Italian Apennines (Fig. 1b). The strain-rates were averaged over a time period (15 ± 3 ka) longer than the timescale of clustered slip. The correlation takes the form of a power law, where strain-rate, $\dot{\varepsilon }$ is related to the elevation, *h*, in the form $\dot{\varepsilon }$ ∝ *h*^*n*^, with *n* = 3.26 (a similar exponent value was determined for the extensional Walker Lane zone in the USA^21^). These authors^18^ considered that *h* contributes to the differential stresses driving the deformation, alongside tectonic forcing, because *h* contributes to the vertical stress. Hence $\dot{\varepsilon }$ ∝ *h*^*n*^ resembles the classic quartz flow law for dislocation creep in quartz shown in Eq. (1)^13^, where, $\dot{\varepsilon }$ is strain-rate, *A* is a material parameter, *fH*_2_O is water fugacity, *m* is the water fugacity exponent, *σ* is the differential stress*, n* is the stress exponent*, Q* is the activation energy*, R* is the ideal gas constant, and *T* is absolute temperature.

1 $$\dot{\varepsilon }=A{{f{H}_{2}}{{{{{\rm{O}}}}}}}^{m}{({\sigma }_{1}-{\sigma }_{3})}^{n}\exp (-Q/RT)$$

The power law form $\dot{\varepsilon }$ ∝ *σ*^*n*^ implies that slip-rates on the brittle faults we study are driven by the strain-rate associated with the underlying viscous shear zones, implying consistency of the finite strain between the brittle and viscous crust over timescales involving multiple large magnitude earthquakes (Fig. 2a). This is consistent with modeling where the strain produced by coseismic slip accrues in a few seconds, and the strain in the middle crust catches up over the entire interseismic period, re-loading the brittle fault^22^. Thus, the strain over time periods containing multiple earthquake cycles, and the strain-rates averaged over time periods longer than postseismic deformation from a single earthquake, are the same in the upper and middle crust^22–24^ (Fig. 2a).

The important question that arises is what would result if the differential stresses within underlying shear zones varied over shorter timescales due to interaction with nearby faults/shear-zones, and were not simply controlled by body forces and regional tectonic forcing acting over the timescale of multiple seismic cycles^18^. As described above, fault slip produces shear strains that deform the surrounding volumes of rock, imposing stresses that alter the timing of slip on other structures in that volume. The same is true of slip across shear zones at depth, because viscous slip will deform the surrounding rocks. The initial elastic deformation imparted onto a shear zone by viscous slip across a neighboring shear zone (Fig. 2c, d), will drive subsequent deformation through viscous creep. Similar deformation behavior is seen in laboratory experiments, in that imposed transient stress changes induce an initial elastic deformation of the rock/mineral sample which is followed by viscous creep^25^. We point out that it is possible to link stress changes produced by slip on natural fault/shear-zone systems to changes in the rates of viscous flow because differential stress is a factor in the flow law for dislocation creep in quartz (Eq. (1)), and it is also a factor in Eq. (2). Equation 2 describes the relationships between shear stresses, the principal stresses and the angle of the fault plane relative to *σ*_1_, and this is used within elastic half-space models to investigate static stress changes^4^ and for brittle failure in general^26^, where *τ* is shear stress, and *β* is the angle between the failure plane and *σ*_1_:

2 $$\tau=\frac{1}{2}\left({\sigma }_{1}-{\sigma }_{3}\right){\sin }2\beta$$

Our key point is that differential stress appears in both Eqs. (1) and (2) implying that elastic interactions between fault/shear-zone structures will alter viscous strain-rates associated with both underlying and neighboring shear zones, a fact that is well-known from existing observations and modeling of postseismic and interseismic deformation after earthquakes^22,23,27^, but has not been used to study the longer timescales and slip-rate changes associated with earthquake clustering.

Our model utilizes localized shear zones because evidence from field exposures of mylonitic shear zones^28^ and numerical modeling of viscous shear zones^29^ indicate that intra-plate mylonitic shear zones are typically hundreds of meters thick or less. Thus, they resemble the localized structures we envisage beneath brittle faults (Fig. 2). The stress transfer model we have developed quantifies stress changes in 3D, allowing the relationship between differential stress changes and viscous strain-rates to be examined in relation to the geometries of neighboring fault/shear-zones.

In summary, the key point that leads to our hypothesis is that slip on localized viscous shear zones and their overlying brittle faults will deform the surrounding volumes of rock, including mineral phases within neighboring viscous mylonitic shear zones, changing differential stress values and hence altering the implied strain-rates given the relationship $\dot{\varepsilon }$ ∝ *σ*^*n*^. The question is whether such interaction can produce changes in slip-rate that replicate observations of temporal clustering of surface faulting earthquakes.

We examine this question in central Italy by attempting to replicate our ^36^Cl-derived findings concerning earthquake clustering through differential stress modeling involving both the viscous shear zones and brittle faults (Figs. 2 and 3).

### Cosmogenic analyses of fault scarps reveal millennial earthquake clusters

The measurements in our study come from the Italian Apennines, a region of extension since 2–3 Ma^30,31^, with active normal faults deforming a pre-existing alpine fold and thrust belt^32,33^. Observations from geodetic, seismological and field-based datasets confirm extension rates of up to ~3 mm/year across the Apennines^34–36^. Historical and instrumental seismicity indicates that large (*M*_w_ 5.5–7.0) magnitude normal faulting earthquakes occur^37,38^ and produce surface carbonate fault scarps^31,39–41^ (Fig. 1d). The surface fault scarps have been preserved since the demise of the last glacial maximum (LGM, 15 ± 3 ka), due to a reduction in erosion rates relative to throw rates at that time^42^ (Fig. 1). These scarps have been studied with in situ ^36^Cl cosmogenic exposure analyses, confirming the post-LGM slope stabilization age and fault slip rate histories that are variable during the Holocene^9,43–45^. In places, dense ^36^Cl sampling has revealed correlation of high slip-rate events with the timing of damaging earthquakes that affected Rome^14^.

We focus on six ^36^Cl sample sites around the Mt. Vettore fault that ruptured in the August–October 2016 sequence, which included Mw 6.2, 6.1 and 6.6 earthquakes^40,46^. We had sampled the faults before the 2016 earthquakes, in the period 2012–2015, to investigate why faults in the region share similar bedrock fault scarp morphologies, but some had ruptured in historical earthquakes whilst others had not, despite being subject to the same regional tectonic stress field. We suspected earthquake clustering might be the cause of such patterns, and quantifiable, prompting our study. In detail, the Mt. Vettore fault ruptured to the surface in the 2016 earthquakes (Fig. 1d), yet paleoseismological studies suggest that before 2016, this SW-dipping active normal fault had not ruptured to the surface for several thousand years, with suggestions of the elapsed time ranging between 1316–4155 years BP^47^, and 1444–1759 years BP^48^. However, during this period, five other nearby faults have ruptured to the surface in damaging historical earthquakes with elapsed times in the order of a few hundred years or less (1349 AD, Fiamignano fault; 1639 AD, Laga fault; 1703 AD, Norcia and Barete faults; 1997 AD Mt. Le Scalette fault; late Holocene, Leonessa fault), revealed by historical accounts, palaeoseismic studies and ^36^Cl studies^14,49–55^. In summary, prior to 2016, the situation was that one fault had not slipped on a millenial timescale, whilst its neighbors had slipped in the same time period (Fig. 5).

We sampled the six faults, sampling parallel to the slip-vector up the fault plane and within shallow (<~1 m) trenches. We constrained the sample sites with geological mapping and topographic surveys (see Supplementary Figs. 1–6). These surveys confirm the exposed fault scarps are formed solely due to tectonic slip and not erosional/depositional processes, because erosional gullies or alluvial fans are not present at these sites, and the footwall and hanging wall cut-offs of the slope that formed at 15 ± 3 ka are parallel and horizontal (see ref. 9 for criteria for choosing a cosmogenic site). We measured ^36^Cl concentrations using accelerator mass spectrometry, and statistically inferred the slip from the ^36^Cl data using a Bayesian Markov chain Monte Carlo (MCMC) approach^14^ (Supplementary Figs. 7–14 and Supplementary Dataset 1; methodology fully described in ref. 14, see Methods for a summary). We model the full scarp height and allow the model to initiate ^36^Cl production (using a Brownian Passage model that allows clustering) when it is needed to replicate the measured values^14^, rather than biasing results by adding an arbitrary pre-exposure value^43,44^ or searching for a single constant peri-glacial fault slip-rate^45^. The results from our least squares solutions and ensembles of least square solutions, as well as highest likelihood solutions that take account of uncertainties and are penalized by priors, show evidence of slip-rate changes that imply temporal earthquake clustering (Figs. 4 and 5 and Supplementary Figs. 7–14). We define earthquake clusters to be periods of rapid slip with slip magnitudes that are too large to be explained by a single earthquake, hence implying that several large magnitude surface rupturing earthquakes and their postseismic episodes have occurred within the cluster. We supplement our ^36^Cl-derived slip histories with published paleoseismology from other nearby faults (Fig. 5).

We have five key findings from our modeling of the ^36^Cl data that help to reveal the cause of the slip-rate changes (Figs. 4 and 5).

1. The slip-rate on the Mt. Vettore fault decreases at ~4 ka and the fault directly across strike from it, the Leonessa fault, accelerates at ~3.5 ka (Fig. 4); palaeoseismology for the Norcia fault may also show acceleration at ~3.5–4.0 ka (Fig. 5). This out-of-phase behavior, revealed by ^36^Cl data on the Leonessa and Mt. Vettore faults (Fig. 4), is the most striking finding in this study, and this has not been reported to date, despite the concentration of studies that surrounded the 2016 earthquake sequence. The faults share similar climate histories, so the differing timings of rapid slip are inconsistent with the notion that fault plane exposure is produced by climate-controlled erosion^16^. We suggest that the out-of-phase slip behavior hints at tectonic interaction between these two structures.
2. Other faults in the region also accelerated at ~3.5 ka. The Laga, Mt. Le Scalette, Fiamignano and Barete faults all show clusters of activity starting at ~3.5 ka in their least squares solution and across their ensembles of least squares solutions (Fig. 5), as well their highest likelihood solutions (Supplementary Figs. 7–12). These faults are along strike from the transect that crosses the Leonessa and Mt. Vettore faults. This finding prompted us to develop a 3D model of fault/shear-zone interaction to include the effects of both across-strike and along strike interaction.
3. Prior to ~4 ka, the Mt. Vettore fault underwent a relatively high slip-rate phase compared to its slip-rate averaged since ~15 ± 3 ka (Fig. 4).
4. Prior to ~3.5 ka, the other faults had slip-rates that were relatively low compared to their 15 ± 3 kyrs average slip rate (Figs. 4 and 5 and Supplementary Figs. 7–12).
5. We note that rapid slip occurred synchronously on the SW and NE flank of the Apennines (e.g., compare slip in the last few thousand years on the Laga and Fiamignano faults; Fig. 5). This finding is inconsistent with the hypothesis that activity migrates, producing clustering, simply due to least-work constraints imposed by spatial changes in dynamic topography^9^.

Our findings are challenging to compare with existing paleoseismic observations^48,51^, because we have sampled different sites along the faults compared to the trenching sites, and slip magnitudes can be difficult to derive from degraded colluvial wedge geometries at trenching sites. Our results are consistent with the palaeoseismic trench site findings in that our results suggest a relatively-long elapsed time for surface faulting on the Mt. Vettore fault prior to 2016, and surface faulting on the other faults in the late Holocene (Fig. 5).

Overall, we suggest that our ^36^Cl results demonstrate clear slip-rate fluctuations through time. The question that arises is what the combined effect of slip-rate changes on brittle faults and implied viscous strain-rate changes at depth have on differential stress values on receiver shear zones, and how these affect strain-rates across the fault/shear-zone system. We investigate this with our modeling described below.

### Calculating the effect of fault interaction on faults and shear zones

We developed a modeling approach to examine fault/shear-zone interaction, linking 3D elastic half-space modeling of the fault slip with 3D elastic half-space modeling of the shear zone slip followed by viscous slip defined by a flow law for dislocation creep (Fig. 3). The modeling includes both across and along strike interactions and details of strike changes along the fault planes^56^. The magnitude of total slip used for the modeling is determined from scaling relationships^15^ and the ^36^Cl slip histories (see Methods), and this slip is applied to the brittle fault and the underlying shear zone (assuming that the horizontal strain and cumulative slip with depth is consistent^22^). The modeling outputs the total differential stress changes over the timescale of each cluster or anti-cluster on each of the 1 × 1 km elements that define the 3D geometry of all the fault/shear-zone systems in the study region^56^.

The modeling approach has a number of simplifying assumptions that we describe here and in the Methods. Our approach is similar to visco-elastic models that concentrate on understanding how postseismic deformation relates to single coseismic slip episodes^22,23,27^, in that we link an upper crust with uniform elastic strength and frictional Coulomb behavior with thermally activated power law creep in the middle crust (15–24 km). However, a key difference is that instead of modeling the effect of single episodes of coseismic slip solely on the underlying shear zone, we model periods of rapid slip lasting several millennia that must include several large magnitude earthquakes and their individual episodes of postseismic deformation, and periods of low or no slip that we refer to as anti-clusters. Another key difference is that we calculate the stress changes produced on neighboring faults/shear-zones as well as on the underlying shear zone. We assume that the most important value of differential stress change is the most negative value on the shallowest portion of the shear zone (i.e., the rate-limiting element). The strain-rate within a shear zone is related to the viscosity^24^. The highest viscosity (and therefore lowest strain-rate) is in the upper part of the shear zone, just below the brittle layer (inset of Fig. 2c). The most negative differential stress change in this section will produce the lowest strain-rate, and thus be the rate-limiting element for deformation within the shear zone. We emphasize that the slip-rates we measure at the surface averaged over clusters will include any postseismic slip from individual earthquakes. We assume that the strain implied by the rapid slip pulse during clusters on brittle faults matches the strain associated with their underlying shear zones (see the spring, dashpot and ratchet inset in Fig. 2a), i.e., the strain-rates in shear zones vary. Furthermore, an important point is that the total differential stress change implied by slip (in a single earthquake or a cluster) is proportional to the total magnitude of slip. Therefore, because we consider total slip (coseismic and any postseismic) in a cluster, postseismic dissipation of differential stress from individual earthquakes is accounted for in our model. Our approach means that it is not necessary to explicitly model strain-rate changes produced by postseismic dissipation after individual earthquakes, although this could be implemented in the future if individual earthquakes could be reliably identified from ^36^Cl data (which we do not believe is possible with current ^36^Cl datasets).

Our approach is to test the following hypothesis; that changes in viscous strain-rate of shear-zones (and thereby changes in slip-rate on overlying brittle faults) are caused by changes in differential stress induced by interactions between neighboring fault/shear-zones. To emphasize the importance of considering changes in viscous strain-rate on underlying shear-zones, we explore whether constant strain-rates are geologically feasible in our dataset. We generate a simple model (Fig. 2) of a shear zone slipping at 1 mm/year (a representative slip rate for the region; Fig. 5 implies fault slip rates of 0.39–2.25 mm/year). This model induces a ~0.01 MPa increase in differential stress at the base of the brittle fault. The longest period of quiescence/anti-cluster in our data is 10 kyr (on the Leonessa fault, Fig. 4). A constant loading rate of 0.01 MPa per year for 10 kyr would result in an additional 100 MPa differential stress. Given that the background differential stress is ~150 MPa at the base of the brittle faults (Fig. 2c), an increase of up to 100 MPa at the base of the brittle crust seems implausibly large for a fault to remain stable/quiescent, especially if fluid pressure changes were also to encourage earthquake rupture. Therefore, constant slip of ~1 mm/year on an underlying shear zone during an anti-cluster seems unlikely, and changes in shear-zone strain-rate have been proposed by others in studies of present-day and palaeoseismic slip-rates^2,8^ and in studies of shear zone microstructures^13,19^. It could be that some of the stress could be dissipated by pressure solution or other small-scale deformation processes, but given the low-rate expected for pressure solution^57^, and uncertainty in the density of active small-scale structures at any given time in the natural crust, it is challenging to quantify whether such stress dissipation is plausible. Therefore we focus our approach by examining whether we can explain our findings of slip-rate changes via differential stress interactions between fault/shear-zones^22,23,27^.

**Fig. 6:**
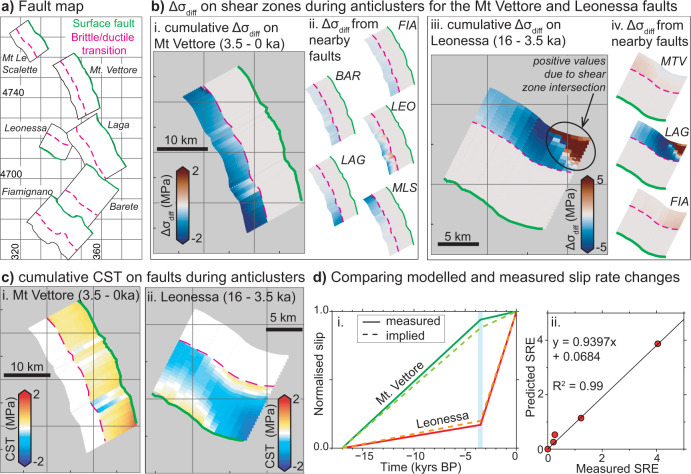
Stress changes and effects on slip rates during periods of quiescence for the Mt. Vettore and Leonessa faults. **a** Fault map showing the locations of the six faults studied. **b** Cumulative differential stress changes (Δ*σ*_diff_) during anti-clusters. (i) On the Mt. Vettore shear zone, which is the sum of (ii) contributions from nearby faults. (iii) On the Leonessa shear zone, which is the sum of (iv) contributions from nearby faults, except the Mt. Le Scalette and Barete faults because Δ*σ*_diff_ are negligible (<±0.05 MPa). The periods of quiescence are shown in the slip histories in Figs. 4 and 5. **c** Cumulative Coulomb Stress Transfer (CST) on the (i) Mt. Vettore and (ii) Leonessa faults during the studied anti-clusters. **d** Comparison between measured slip histories from ^36^Cl and slip histories inferred from differential stress changes and the quartz flow law. (i) Slip histories for the Mt. Vettore and Leonessa faults normalized to the total measured slip. (ii) Slip Rate Enhancement (SRE) values are calculated relative to the long-term (15 ± 3 kyr rate) slip rate, where SRE <1 implies a slowing of slip and a reduction in activity. The similarity between measured and implied slip histories suggests the approach we use, combining stress changes with quartz flow laws, to generate the implied slip histories replicates the natural system.

To quantify interactions between the faults, shear zones, and neighboring fault/shear-zones, and their effect on strain-rates, slip-rates and clustering, we extracted the amount of slip on each fault in the time period from ~3.5 ka to 2015 AD, and prior to ~3.5 ka (Figs. 4 and 5). We modeled both the Coulomb stress transfer (CST)^5^ and differential stress transfer (Δ*σ*_diff_) onto receiver fault/shear-zones implied by the amount of slip derived from the ^36^Cl modeling in each time period (Fig. 6, Supplementary Figs. 15–18 and Supplementary Dataset 2). We concentrate our analysis on the Mt. Vettore and Leonessa faults, because these faults are located centrally in the study area and receive stress from slip on both along-strike and across-strike structures that we can constrain with ^36^Cl and paleoseismic data^51,58^ (Figs. 4 and 5). The calculations reveal stress-loading histories during temporal earthquake anti-clusters, on the Mt. Vettore and Leonessa faults and underlying shear zones (Fig. 6, Supplementary Figs. 15–18 and Supplementary Dataset 2). We discuss the results for faults and shear zones separately.

For faults, we do not find a consistent pattern of increasing or decreasing CST during anti-clusters. For the Mt. Vettore fault, we find that the CST from slip on neighboring fault/shear-zones became mostly positive during its quiescence from ~3.5 ka to present (Fig. 6ci), before it ruptured in 2016^59^. This makes sense because an earthquake after a relatively-long elapsed time is perhaps intuitively expected because faults will be loaded through time by body forces and far-field tectonic forces^60^, and CST may positively load the fault^61^. However, this intuitive view breaks down for the Leonessa fault, because the CST became increasingly negative during its low slip-rate time period from 17 to ~3.5 ka (Fig. 6cii). Despite the negative CST, the Leonessa fault did not cease activity, with ^36^Cl data indicating an accumulation of 6.5 m slip between ~3.5 ka to present, with historical constraints narrowing this to 3.5 to 0.7 ka, proving it is a Holocene active fault^54^. Overall, it appears that CST on brittle faults does not directly explain why brittle faults experience anti-clusters and then rupture, as the loading can be positive or negative due to fault interaction.

In contrast, for shear zones we do find a consistent pattern of stress loading during anti-clusters. During the two anti-clusters we study, the differential stress changes for shear zones are mostly negative. The Mt. Vettore shear zone experienced a stress reduction of up to -1.8 MPa just below the brittle-viscous transition between 3.5 ka and 2015 AD (Fig. 6bi). The Leonessa shear zone experienced a stress reduction of up to −3.4 MPa just below the brittle-viscous transition between 17 and 3.5 ka (Fig. 6bii). These values are significant given that we expect the background differential stress in shear zones in the middle crust to be only ~10 MPa, and essentially constant over the ~15–24 km depth range, from investigations of exhumed extensional shear zones^19^, so changes of −1.8 to −3.4 MPa would reduce the differential stresses produced by the ambient conditions by 18–34%. Values of differential stress are negative everywhere on the shear zones except where they intersect at depth in the model (Fig. 6). If our assumption is correct, and these are rate-limiting elements, modeling their subsequent deformation will be the key to understanding how strain is transferred upwards onto the overlying brittle faults.

Thus, our finding that differential stress change in the underlying shear zones was negative when both overlying faults had very low slip-rates (anti-clusters) prompted us to investigate whether the magnitudes of differential stress reduction generate strain-rate changes comparable to our findings from ^36^Cl.

### Calculating changes in viscous strain-rate implied by differential stress changes produced by fault/shear-zone interaction

To calculate the implied change in strain-rate for each shear zone within the two anti-clusters, we input the reductions of differential stress into Eq. (1), using appropriate values for other variables^13^ (Fig. 3 and Supplementary Dataset 3). Assuming the patch with the largest stress decrease is the rate-limiting element^24^, a key assumption in our approach, it is implied that strain-rates would have decreased from 1.5 × 10^−16^ s^−1^ (the strain-rate before a stress change, see Supplementary Dataset 3) to 7.7 × 10^−17^ s^−1^ on the Mt. Vettore shear zone between 3.5 ka and 2015 AD, and to 3.7 × 10^−17^ s^−1^ on the Leonessa shear zone between 17–3.5 ka (see Supplementary Dataset 3). Thus, both shear zones were still active during periods of earthquake quiescence, albeit with reduced strain-rates, giving rise to long recurrence intervals. Even with reduced strain-rates, the shear zone loading is eventually able to overwhelm the impact of negative stress changes on the brittle faults, generating earthquakes that signify the end of an anti-cluster.

To compare the effect of the implied strain-rate changes with our ^36^Cl measurements of the natural system, we converted the strain-rates in the shear zones into implied slip-rates on the overlying brittle faults and compared them with the observed slip-rates (Fig. 6d). We used the slip measured over the total time period constrained with ^36^Cl as a measure of the long-term (15 ± 3 kyr) slip-rate^18^. We compare these long-term slip-rates with short-term slip-rates during clusters/anti-clusters by calculating slip-rate enhancement factors (SRE, calculated by dividing the short-term slip rate by the 15 ± 3 kyr slip rate) that describe how much the slip-rates were enhanced (SRE >1) or impeded (SRE <1) compared to the long-term slip-rates (Fig. 6bii). SRE values range between <1 to >4 in both the measured and implied slip-rate datasets. We find that there is good agreement between the implied slip histories and those measured from ^36^Cl analyses (Fig. 6d and Supplementary Dataset 3). This implies that our relatively simple model, with the assumptions stated above, can quantitatively replicate key slip-rate findings from our investigation of the natural system, providing insight into the processes that drive earthquake clustering and anti-clustering.

By combining surface findings (^36^Cl-derived slip histories) with stress modeling and rock mechanics experimental results^13^, for the example described herein, we suggest that interaction between neighboring fault/shear-zones may be the dominant control on temporal earthquake clustering.

## Discussion

Earthquake clustering confounds our ability to understand and quantify seismic hazard because the greater the unknown aperiodicity in recurrence intervals in fault-based time-dependent hazard assessments, the greater the uncertainty that will need to be communicated probabilistically with regard to recurrence of expected ground accelerations within stated time periods^62^. Greater uncertainty may lead to reluctance to implement costly mitigation strategies and greater challenges in effective communication that triggers action amongst those at risk. One approach to explain the aperiodicity is to suggest that the processes that control slip are multiple, complex, interacting, and difficult to quantify, and the system may be considered as approaching random behavior^63^. However, the key implication herein is that, instead, earthquake clustering appears to have a dominant, quantifiable cause for the example we study, and is therefore not random. Our results suggest that viscous shear zones slow or accelerate due to changes in differential stress produced by slip on nearby viscous shear zones and brittle faults. Our results suggest that upper crustal brittle fault interaction^64^, or least-work constraints imposed by dynamic topography^9^ are unlikely to be the sole controls responsible for earthquake clustering. Our interpretation, where shear zone strain-rates change due to stress transfer altering the differential stress, may be linked to suggestions that tectonic strain is stored during anti-clusters^2,65^, and/or may be linked to the mechanism by which microstructural evolution leads to shear zone strengthening during anti-clusters if microstructural changes occur during strain-rate fluctuations^6^. Clearly, more work is needed to examine other viscous flow laws, more complicated shear zone geometries, different fault arrays and interaction over shorter timescales. However, the links we have made between geomorphic offsets, cosmogenic dating of fault scarps, calculations of stress transfer, and viscous flow laws, provide important new insights into continental mechanics and seismic hazard that go beyond what can be achieved by simply studying instrumental seismicity. In particular, our results suggest that we should expect slip-rate and strain-rate changes through time on the timescale of earthquake clustering, as these are the natural consequence of fault and shear zone interactions. These slip-rate changes will alter earthquake recurrence rates, and therefore the calculated Tmean (inter-event time^64^) and the Coefficient of Variation (CV, the standard deviation of inter-event times divided by Tmean^66^) during and across clusters and anti-clusters will be different. As key inputs for fault-based seismic hazard assessments, we suggest that different values of Tmean and CV within clusters and anti-clusters should be considered in seismic hazard calculations, although, exactly how slip-rate fluctuations are incorporated into PSHA for both data-rich and data-poor regions remains an open question that requires further study. Our approach warrants further study and we suggest that an independent test of our model will require calculations of stress change due to slip within time periods with precise time constraints such as we provide herein. Such studies will improve our ability to use values of slip-rate variability and aperiodic earthquake recurrence within fault-based probabilistic seismic hazard assessments^6^.

## Methods

### Inversion of slip histories from ^36^Cl cosmogenic dating

Sites for cosmogenic sampling from limestone bedrock faults planes are carefully selected to ensure that the scarps are formed solely by tectonic exhumation (see Supplementary Figs. 1–6 which describes the characteristics of each sample site, and Supplementary Table 1 which gives the site parameters required for ^36^Cl modeling). A good site will have parallel hanging wall/footwall intersections with the fault plane, a smooth lower slope on the hanging wall devoid of erosional or depositional features, and will avoid active gullies or other erosional features present on the footwall or fault plane. 15 × 5 × 2.5 cm sized samples of fault plane were taken parallel to the slip vector measured from frictional wear striations. These samples were prepared following the approach of refs. 9,67 and were analyzed with AMS to determine the concentrations of ^36^Cl in each sample (Supplementary Dataset 1). The concentration of ^36^Cl increases up the fault plane as the length of time of exposure increases. We used the Bayesian MCMC code of ref. 14 to inverse model the slip history from measured concentrations of ^36^Cl (results of the modeling are shown in Supplementary Figs. 7–12). This code searches for the probability distribution of the slip history conditioned on the measured data, and as an outcome identifies a slip history of least-squares and highest likelihood fit, while allowing a high flexibility of the magnitude and timings of slip events, uncertainties in the density of the colluvium and ^36^Cl production factors, and timing of ^36^Cl initial production. We have also iterated inputs, such as the total slip across the scarps (Supplementary Fig. 13), and find that the strain-rate and SRE results are relatively insensitive to uncertainty in these values. We also show that sample spacings on the fault planes we achieved are adequate to resolve the slip-rate changes we claim. We do this by progressively degrading the dense sampling for the Fiamignano fault to a point where two well-constrained historical earthquake sequences resolvable with the full data disappear (Supplementary Fig. 14). The full approach to the statistical modeling of slip histories using the ^36^Cl data is described in detail in ref. 14.

### Assumptions used in modeling slip on fault/shear-zones

1. We assume that shear zones have the same dip as overlying brittle faults^5,18,59,68,69^. We make this assumption because where the structure of the middle/lower crust beneath areas of extension has been clearly imaged with high quality seismic reflection data (e.g., the DRUM profile offshore N. Scotland^70^ and the Viking Graben of the North Sea^71^), shear zones have relatively steep dips that are similar to those of overlying brittle faults. For the northern Apennines, Italy, deep seismic reflection images exist for the middle/lower crust^72–74^, and shear zones with relatively shallow dips have been interpreted. However, further south, seismic quality is in places relatively poor, especially where thick carbonates dominate the surface geology (e.g., parts of deep seismic reflection line CROP 11^75^; Fig. 1). Low-angle extensional detachments/shear zones have been proposed to explain low-angle reflections along the deep seismic reflection line CROP 3 only where arenaceous turbidites outcrop at the surface, which is ~70–100 km to the NW of the area we study. The interpretation of low-angle detachments is also debated due to the lack of low-angle nodal planes for microearthquakes located along the low-angle reflection(s)^74^, and the fact that Alpine nappe geometries exhibit a transition from metamorphic Tuscan Nappe geometries in the WSW to Miocene arenaceous turbidites in the ENE, implying that the low-angle reflections dipping toward the ENE may be due to the general ENE dip to the Alpine geology of the nappe pile rather than a primary seismogenic detachment^75^. The area we study is closer to the line of CROP 11 (Fig. 1), which is dominated by carbonates at the surface in the extensional area of the Apennines, and low-angle reflectors are less prominent or absent compared to on CROP 3. Hence, we prefer to use the structural style imaged in areas with clear images of the middle/lower crust and choose to model shear zones that have the same dip as overlying brittle faults; future studies can investigate the implications of modeling low-angle detachments if they prove necessary.
2. We assume that the shear zones are relatively localized so we can utilize an elastic half-space model to model stress changes on receiver fault/shear-zones. We have chosen this geometry (e.g., ref. 12), because numerical modeling of the scaling of viscous shear zones with depth-dependent viscosity and power-law stress-strain dependence imply that shear zones in the viscous crust are 1.7–3.5 km in thickness for a wide variety of parameter choices^29^. This is consistent with the *T* ∝ *D* scaling relationships between shear zone thickness (*T*) and displacement (*D*) for exhumed shear zones from a variety of magmatic and metamorphic rocks^28^, which imply that if shear zones exhibit similar offsets to their overlying brittle faults, the 1–2 km offsets of pre-rift strata measured at surface in the area we study^31^ would be consistent with shear zone thicknesses of only 1–2 km. This suggests that localized shear zones in the middle crust^12^ and elastic half-space models of creep at depth may be widely applicable^29^, prompting the geometries we utilize in Fig. 2.
3. We assume the shallowest parts of the shear zones have the highest resistance to deformation^24^, and therefore control the rate at which shear strain and differential stress are passed upwards onto the overlying brittle faults. We assume this because, as mentioned above^24^, shear zones will have a depth-dependent rheology, controlled by the increase in temperature with depth. This translates into a depth-dependent viscosity, which for a geothermal gradient of 25 K/km, implies an effective viscosity varying from ~10^22^ Pa S at ~15 km depth to ~10^19^ Pa S at 30 km depth^24^. Our model allows us to calculate the changes in differential stress over the depth range of 15–20 km and deeper, and convert this into expected strain-rate changes, and how these vary with depth, by including depth variation in lithostatic pressure and water fugacity in our calculations (Supplementary Datasets 2 and 3). However, we consider the region of highest resistance to deformation near the top at the shear zone to be the rate-limiting element in passing shear strain and differential stress upwards onto the overlying brittle faults. We use the minimum value in the depth range of 15–16 km as input to the quartz flow law, appropriate for the depth of viscous flow in the area we study^18^. Future studies can explore the implications of using depth variation in viscosity and strain-rate, and the notion of a rate-limiting element if thought appropriate.
4. We assume that the rate of slip on the shear zone matches that of the overlying fault (Fig. 2a), supported by the data in Fig. 1^18^, and modeling of the links between brittle surface slip and deeper ductile flow where the total strain accommodated by slip on brittle faults over many seismic cycles is matched at depths where viscous deformation occurs^22,23,27^.
5. We assume that slip-rates at the surface over numerous earthquake cycles implied by our modeling ^36^Cl data includes any localized postseismic afterslip following individual earthquakes. This implies that the slip-rate variations we study should be analyzed over timescales longer than that of individual postseismic slip episodes.

### Modeling Coulomb stress changes

Non-planar strike-variable fault geometries are built as a series of rectangular elements^56^ that are ~1 km^2^. The geometry of the faults is based on extensive field data collected from limestone bedrock fault scarps in the central Apennines^5,31,35,76–81^. These strike-variable fault geometries are utilized in Coulomb 3.4^61^ to model Coulomb stress changes associated with earthquakes and slip on underlying shear zones. The brittle-viscous transition is assumed to be at 15 km depth and we model the portions of shear zones that extend from 15–24 km depth, as this is the depth range over which viscous flow will initiate^18^, and this is also the depth range that will have the highest resistance to deformation and hence the rate-limiting elements (i.e., the elements with the minimum stress) for passing shear strains upwards onto brittle faults^24^. Altering the depth of the modeled brittle viscous transition will not alter the sense (positive or negative) of deformation rates changes. For each fault, a characteristic earthquake magnitude is calculated using the relationship between fault area and magnitude^15^. A simple concentric slip distribution is calculated, assuming 40% of the maximum slip at depth reaches the surface, and the maximum slip is iterated to match the earthquake magnitude. The 40% assumption is based on iterating this value to closely match the ratios between (1) average subsurface displacement and maximum surface displacement and (2) average subsurface displacement and average surface displacement^15^ (0.76 and 1.32 modal values respectively), which also matches the findings of others^82^. We have been unable to exactly match the modal values, however the values reported herein are within the variability reported^15^. The values used to calculate the characteristic magnitude are given in Supplementary Table 2.

The contribution of each structure to the CST on the brittle faults is shown in Supplementary Figs. 15–18 and Supplementary Dataset 2. The annual magnitude of slip on underlying shear zones is calculated from the Holocene throw profiles measured through fieldwork, as these are suggested to be equivalent^43^.

### Calculating differential stress changes

Coulomb stress changes are defined as $\Delta {{{{{\rm{CST}}}}}}=\Delta \tau+\mu \Delta {\sigma }_{n}$^83^, where $\Delta \tau$ is the change in shear stress, *μ* is the coefficient of friction (herein 0.4 is used^56^) and $\Delta {\sigma }_{n}$ is the change in normal stress. The shear stress can be defined as $\tau=\frac{1}{2}\left({\sigma }_{1}-{\sigma }_{3}\right){\sin }2\beta$^4^ where $\left({\sigma }_{1}-{\sigma }_{3}\right)$ is the differential stress and *β* is the angle between ${\sigma }_{1}$ and the fault plane. In the central Apennines, normal faulting is dominant and therefore we assume ${\sigma }_{1}$ is vertical. Therefore $\beta=90-\theta$ where *θ* is the dip of the fault. We have calculated the differential stress using the equations above and the shear stress calculated from Coulomb 3.4. The differential stress is calculated for each 1 × 1 km rectangular fault patch for the brittle and viscous portions of the faults. The conversion between sig_reverse (direct output from Coulomb 3.4, which is shear stress on the fault plane) and differential stress is given in Supplementary Dataset 2.

### Calculating change in strain-rates

Viscous deformation via dislocation creep, derived from laboratory experiments, is given by the following equation^13^: $\dot{{{{{{\rm{\varepsilon }}}}}}}={{{{{\rm{A}}}}}}{{{{{{\rm{f}}}}}}}_{{{{{{{\rm{H}}}}}}}_{2}{{{{{\rm{O}}}}}}}^{{{{{{\rm{m}}}}}}}{{{{{{\rm{\sigma }}}}}}}^{{{{{{\rm{n}}}}}}}{{{{{{\rm{e}}}}}}}^{\frac{-{{{{{\rm{Q}}}}}}}{{{{{{\rm{RT}}}}}}}}$, where $\dot{\varepsilon }$ is the strain-rate, *A* is a material parameter, ${{{{{{\rm{f}}}}}}}_{{{{{{{\rm{H}}}}}}}_{2}{{{{{\rm{O}}}}}}}^{{{{{{\rm{m}}}}}}}$ is the water fugacity, *σ* is the differential stress, *n* is the stress exponent, *Q* is the activation energy, *R* is the ideal gas constant and *T* is the temperature. For the dislocation creep of wet quartz, the following constant values are used: *A* = 6.31e−12 MPa/s, *Q* = 35 kJ/mol^13^, *R* = 8.31 m^2^ kgs^−2^ K^−1^ mol^−1^, *n* = 3.26^18^, T = 710K/440 °C^18^, ${f}_{{{{{{\rm{{H}}}}}_{2}O}}}^{m}$ = 110 MPa (calculated given T = 440 °C and pressure = 0.4 GPa @15 km depth using the online fugacity calculator^84,85^). We choose this flow law for the following reasons: (1) dislocation creep mechanisms are common in natural quartz-bearing shear zones that dominate middle continental crust at the temperature and pressure range described here^37^; (2) the chosen flow law^13^ considers the effect of water fugacity and is relatively well-constrained via comparison to naturally deformed rocks; (3) the use of this flow law allows consistency with previous studies in this region from which we take the stress exponent^18^, and with other visco-elastic models of postseismic deformation after earthquakes^22,23,27^. We implement the calculations using Supplementary Dataset 3 and following the method detailed in Fig. 3. Although the published flow law^13^ uses *n* = 4, we substitute *n* = 3.26 as derived for the Apennines region^18^. This has little effect on the resulting strain-rate, which is the same order of magnitude at 10 MPa differential stress. The background value of differential stress is taken to be 10 MPa as values across this depth range are thought to be relatively uniform^19^. The change in differential stress is calculated from the stress modeling. Sensitivity to the chosen values for differential stress and stress exponent are shown in Supplementary Fig. 19. Sensitivity to overestimating or underestimating the amount of slip across the scarps for strain-rates is shown in Supplementary Fig. 20. We converted the implied strain-rates for the shear zones into implied slip-rates and slip-rate changes for the overlying brittle faults by using (1) the ratio of strain-rates before and after the rate changes, and (2) the slip-rates over the entire period constrained in terms of timing from ^36^Cl, and offset using scarp profiles at the surface (Supplementary Dataset 3). These 15 ± 3 kyr slip-rates were multiplied by the ratio of strain-rates before and after the rate changes, and amounts of slip were recovered before and after slip-rate changes, by multiplying the ratio-modified slip-rates by the time periods in question. We used these values to compare measured and implied SRE values. We also show that implied earthquake recurrence intervals for 1 m slip events (typical of the region) are of reasonable duration (a few millennia from paleoseismology^47,51^), given the values we input into the quartz flow law, by calculating the recurrence intervals for 1 m heave events, given that we can measure the across strike distance for the region, and can calculate heave rates before and after strain-rate changes assuming faults and shear zones dip at 45°. Supplementary Dataset 3 shows that recurrence intervals for 1 m heave events change from ~3.6 kyrs to ~10–19 kyrs during anti-clusters, comparable in terms of order of magnitude to values from paleoseismology.

## Supplementary information

Supplementary Information

Description of Additional Supplementary files

Supplementary Dataset 1

Supplementary Dataset 2

Supplementary Dataset 3

## Data Availability

The cosmogenic data utilized in study are published online in the British Geological Survey repository and is freely available for download at https://www.bgs.ac.uk/services/ngdc/accessions/index.html#item128345. The samples for cosmogenic analysis were collected responsibly with support from local geologists. The processed ^36^Cl data and strain rate calculations are provided in the Supplementary Figures and Data.

## Code availability

The code to model 3D strike-variable fault planes^56^ for use in Coulomb 3.4 is available from https://github.com/ZoeMildon/3D-faults/releases/tag/v1.0. The code to model interseismic loading from underlying shear zones is available from https://github.com/ZoeMildon/3D-faults-shearzones (10.5281/zenodo.7149495). The code used to invert for slip histories from ^36^Cl measurements^14^ is available from https://github.com/beckjh/bed36Cl (10.5281/zenodo.1402093).
