# Supplementary material for: Surface faulting earthquake clustering controlled by fault and shear-zone interactions
Source: Nat Commun. 2022 Nov 21;13:7126. doi: 10.1038/s41467-022-34821-5 (PMC9681762; doi:10.1038/s41467-022-34821-5)
Supplement: Supplementary file 1 — Supplementary Information [file 41467_2022_34821_MOESM1_ESM.pdf]

Supplementary information for

Surface faulting earthquake clustering controlled by fault and shear-zone interactions

Zoë K. Mildon[1,*], Gerald P. Roberts[2], Joanna P. Faure Walker[3], Joakim Beck[4], Ioannis Papanikolaou[5], Alessandro M. Michetti[6,7], Shinji Toda[8], Francesco Iezzi[9], Lucy Campbell[10], Kenneth J.W. McCaffrey[11], Richard Shanks[12], Claudia Sgambato[2], Jennifer Robertson[2], Marco Meschis[13], Eutizio Vittori[14],

1. School of Geography, Earth and Environmental Sciences, University of Plymouth, Drakes Circus, Plymouth, PL4 8AA, UK
2. Department of Earth and Planetary Sciences, Birkbeck, University of London, London, WC1E 7HX, UK
3. IRDR, University College London, Gower Street, London, WC1E 6BT, UK.
4. Computer, Electrical and Mathematical Sciences and Engineering, 4700 King Abdullah University of Science and Technology (KAUST), Thuwal 23955-6900, Kingdom of Saudi Arabia
5. Laboratory of Mineralogy and Geology, Department of Natural Resources Development & Agricultural Engineering, Agricultural University of Athens, 11855 Athina, Greece
6. Dipartimento di Scienza ed Alta Tecnologia, Università degli Studi dell'Insubria, Como, Italy.
7. Istituto Nazionale di Geofisica e Vulcanologia, Sezione di Napoli Osservatorio Vesuviano, Via Diocleziano 328, 80124 Naples, Italy
8. International Research Institute of Disaster Science, Tohoku University, Sendai, Japan.
9. DiSTAR - Dipartimento di Scienze della Terra, dell'Ambiente e delle Risorse, University of Naples "Federico II", Italy.
10. School of Environmental Sciences, University of Hull, Hull, HU6 7RX, UK
11. Department of Earth Sciences, University of Durham, Durham, DH1 3LE, UK.
12. Scottish Universities Environmental Research Centre, Glasgow, G75 0QF UK
13. Istituto Nazionale di Geofisica e Vulcanologia, Sezione di Palermo, via Ugo La Malfa, 153, 90146 Palermo, Italy
14. CNR, Institute of Geosciences and Earth Resources, via La Pira 4, 50121 Florence, Italy

* Corresponding author: Zoë Mildon, zoe.mildon@plymouth.ac.uk

This file includes:

Supplementary Figures 1 – 20

Supplementary Tables 1 – 2

Supplementary References

Fiamignano fault Lat. 42.272969°, Long. 13.114854°, Elevation 1150m.

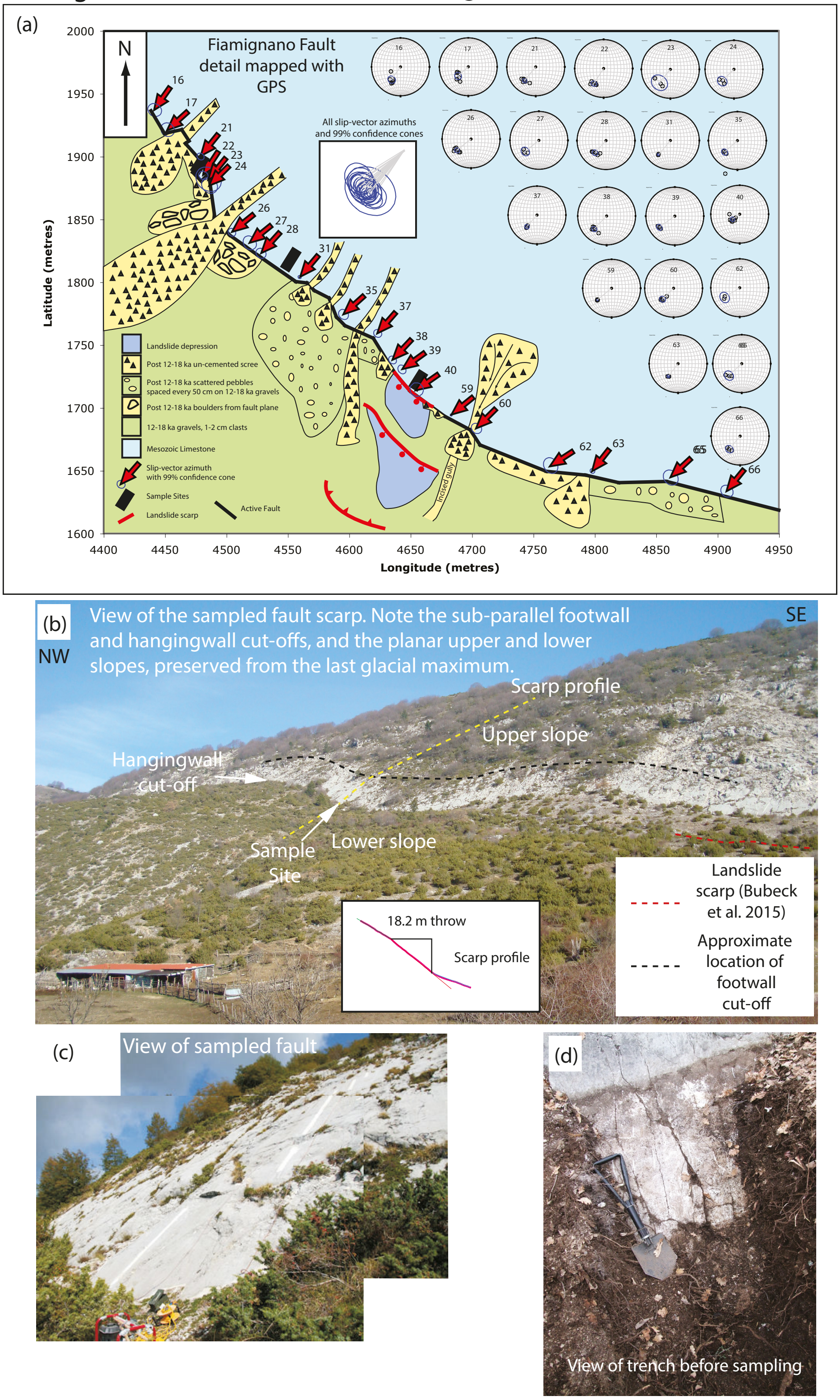

Supplementary Figure 1 – Site characterisation for ^{36}Cl sample site on the Fiamignano fault. a) Structural and geomorphic map of the area around the sample site (previously published in ref.[1]), b) View onto the sample site with the key geomorphic features annotated, c) Close up view of the sampled fault plane, d) View of the fault plane in the trench prior to sampling. All field photos were taken and annotated by the authors.

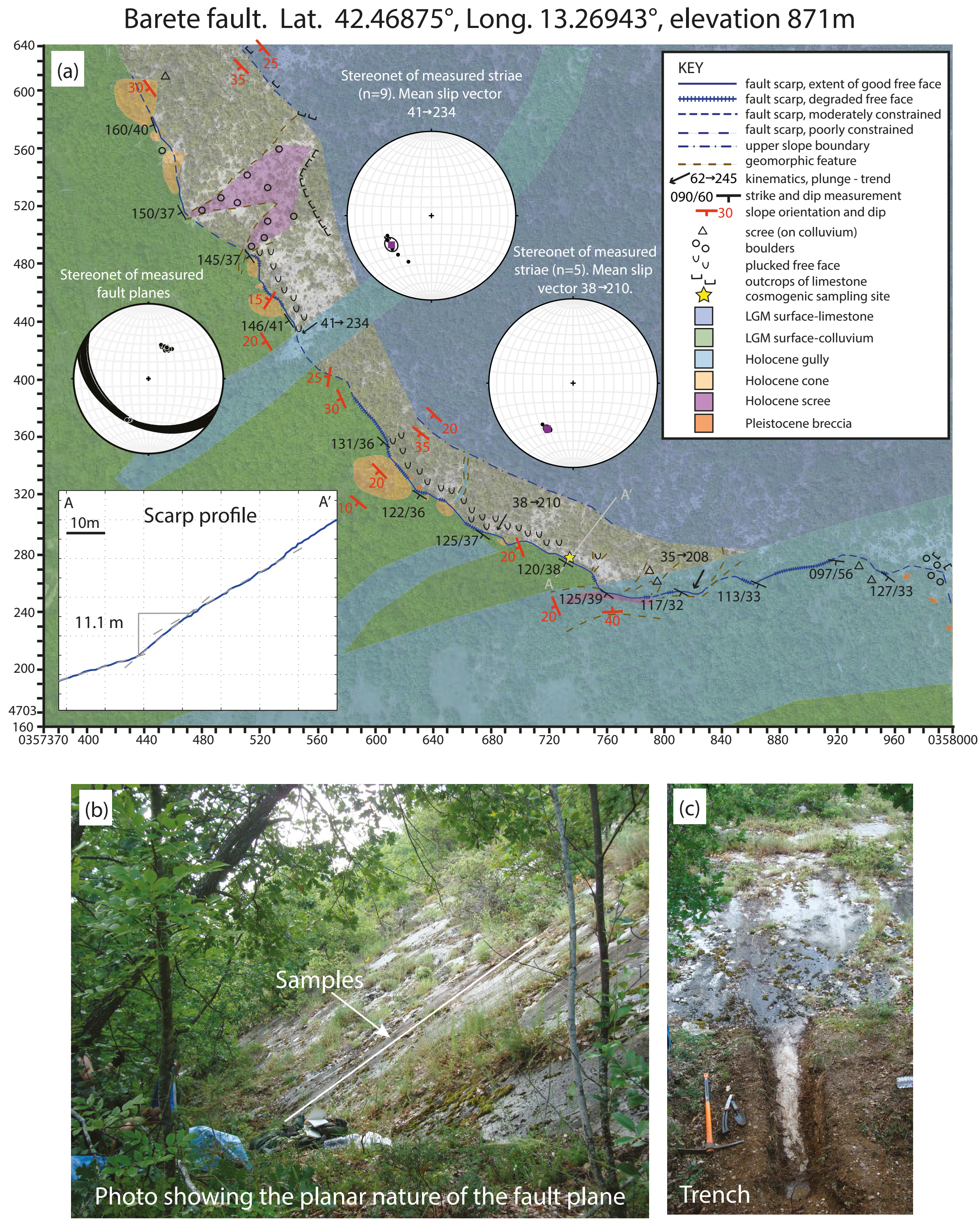

Supplementary Figure 2 - Site characterisation for ^{36}Cl sample site on the Barete fault. a) Structural and geomorphic map of the area (overlayed on Google Earth imagery) around the sample site (previously published in ref.[2]), b) View onto the sample site showing the planar nature of the fault plane, c) View of the fault plane in the trench prior to sampling. All field photos were taken and annotated by the authors.

Mt. Le Scalette fault. Lat. 43.06345°, Long. 12.91527°. Elevation 975m

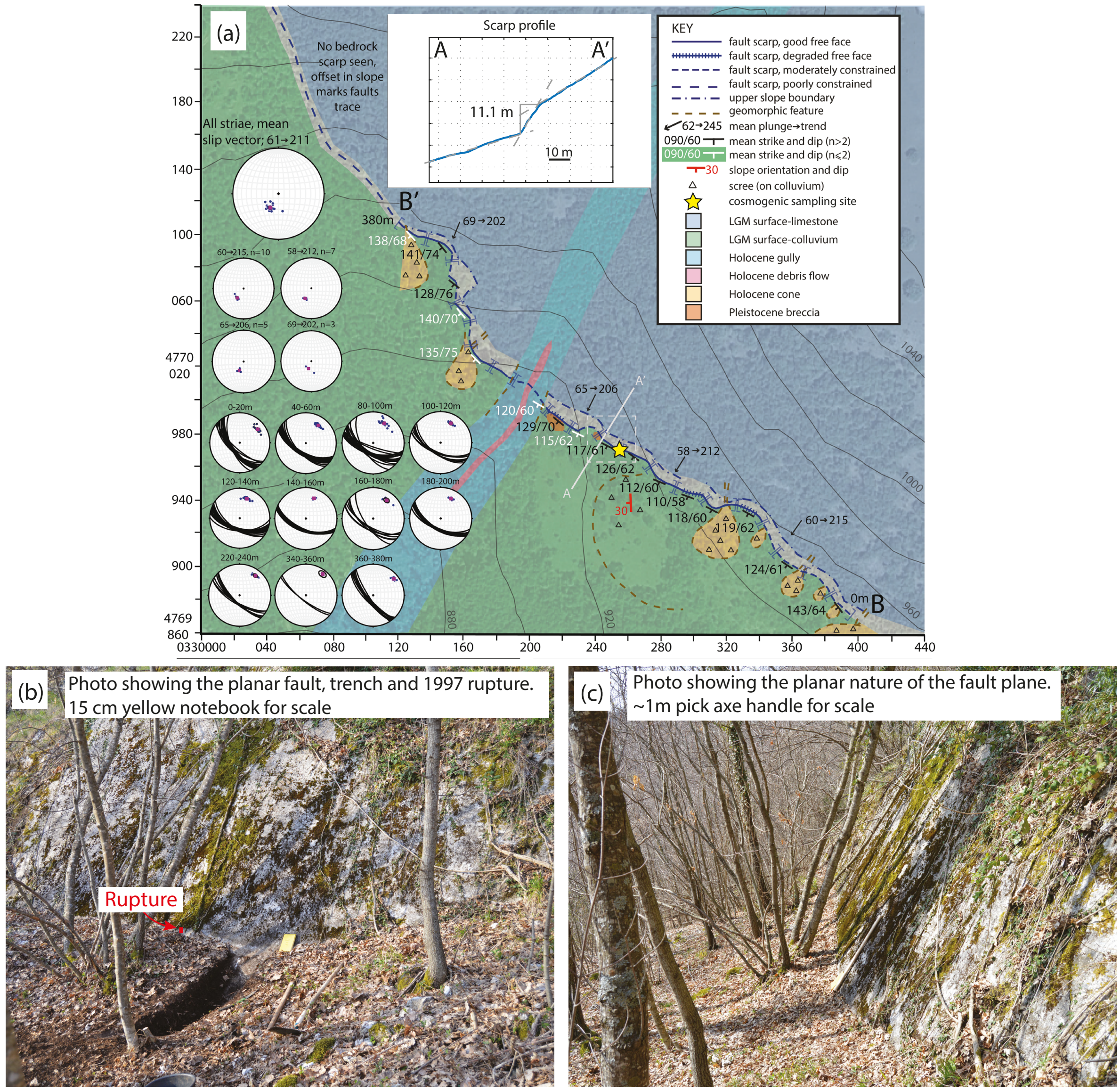

Supplementary Figure 3 - Site characterisation for ^{36}Cl sample site on the Mt. Le Scalette fault. a) Structural and geomorphic map of the area (overlayed on Google Earth imagery) around the sample site (previously published in ref.[2]) b) View onto the sample site showing the 1997 coseismic rupture and trench, c) View along the sample site showing the planar nature of the fault plane. All field photos were taken and annotated by the authors.

Leonessa Fault. Lat. 42.56138°, Long. 12.95964°, Elevation 1063 m

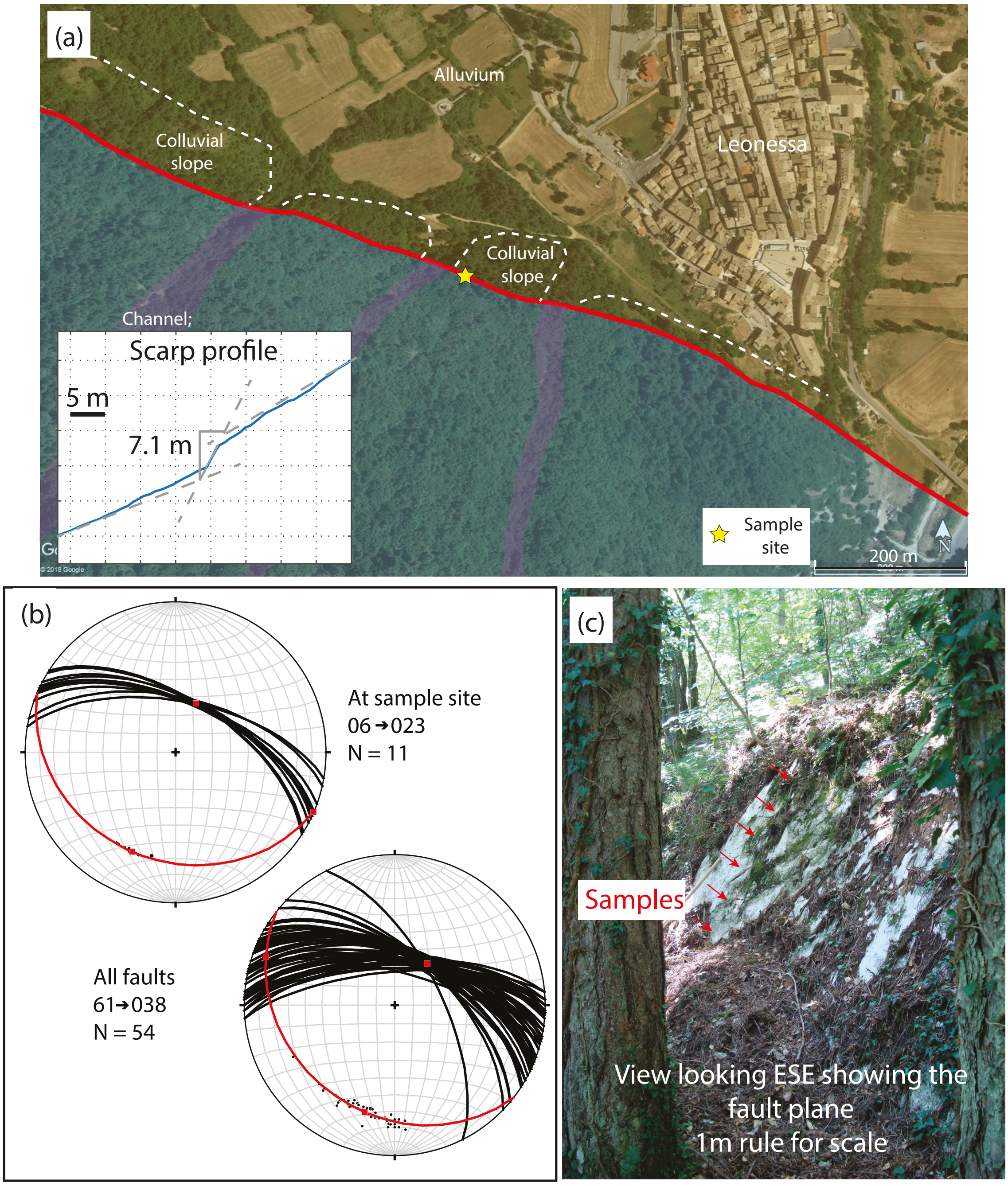

Supplementary Figure 4 - Site characterisation for ^{36}Cl sample site on the Leonessa fault. a) Geomorphic map of the area around the sample site mapped from Google Earth imagery and in the field, b) Stereonets of structural data from the sample site and along the mapped fault, c) View onto the sample site showing the planar nature of the fault plane and the location of samples. All field photos were taken and annotated by the authors.

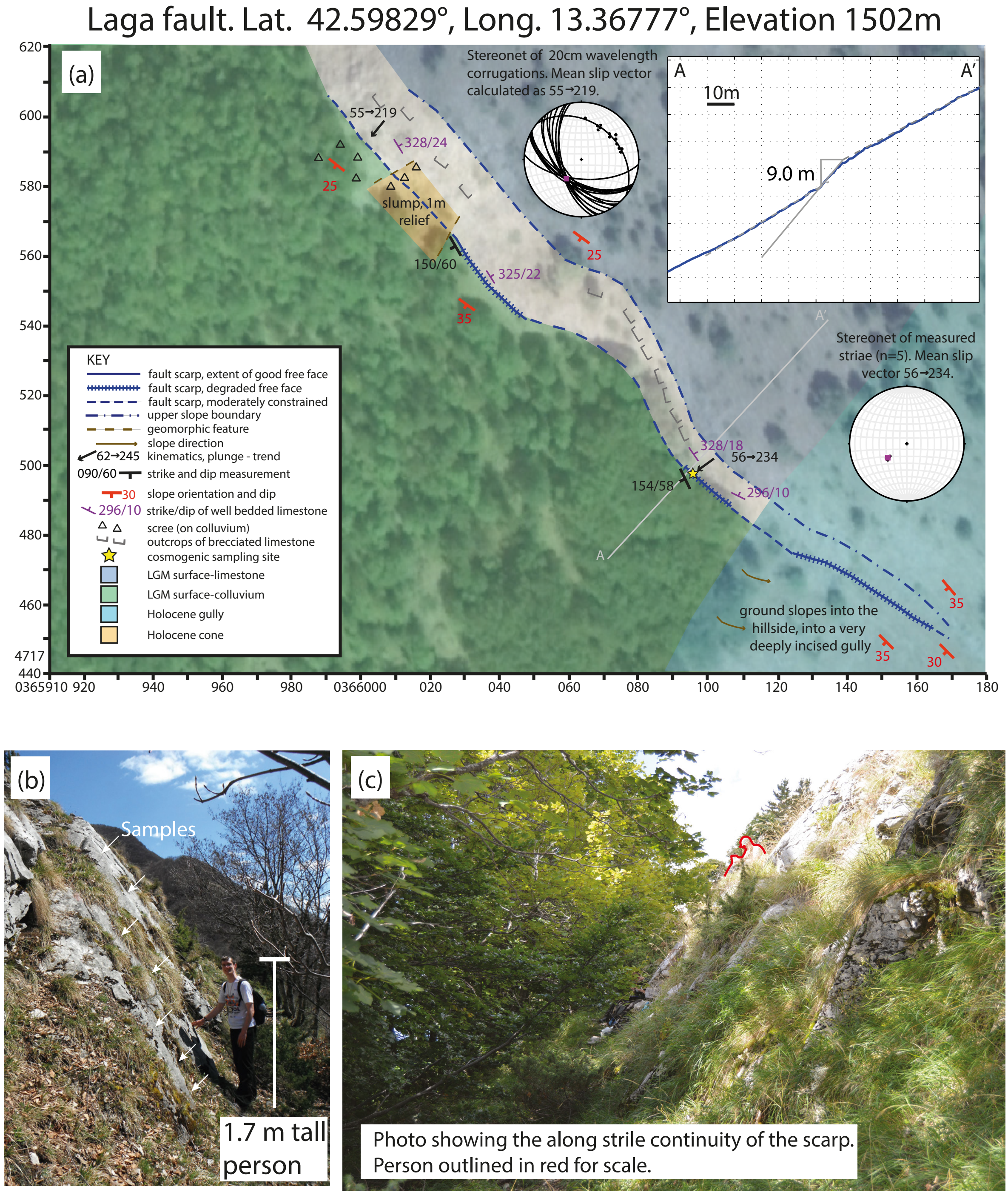

Supplementary Figure 5 - Site characterisation for ^{36}Cl sample site on the Laga fault. a) Structural and geomorphic map of the area (overlayed on Google Earth imagery) around the sample site (previously published in ref.[2]), b) View onto the sample site showing the planar nature of the fault plane and sample locations, c) View along the fault at the sample site showing along-strike continuity of the scarp. All field photos were taken and annotated by the authors.

Mt Vettore fault. Lat. 42.86695°, Long. 13.22707°, Elevation 1800m.

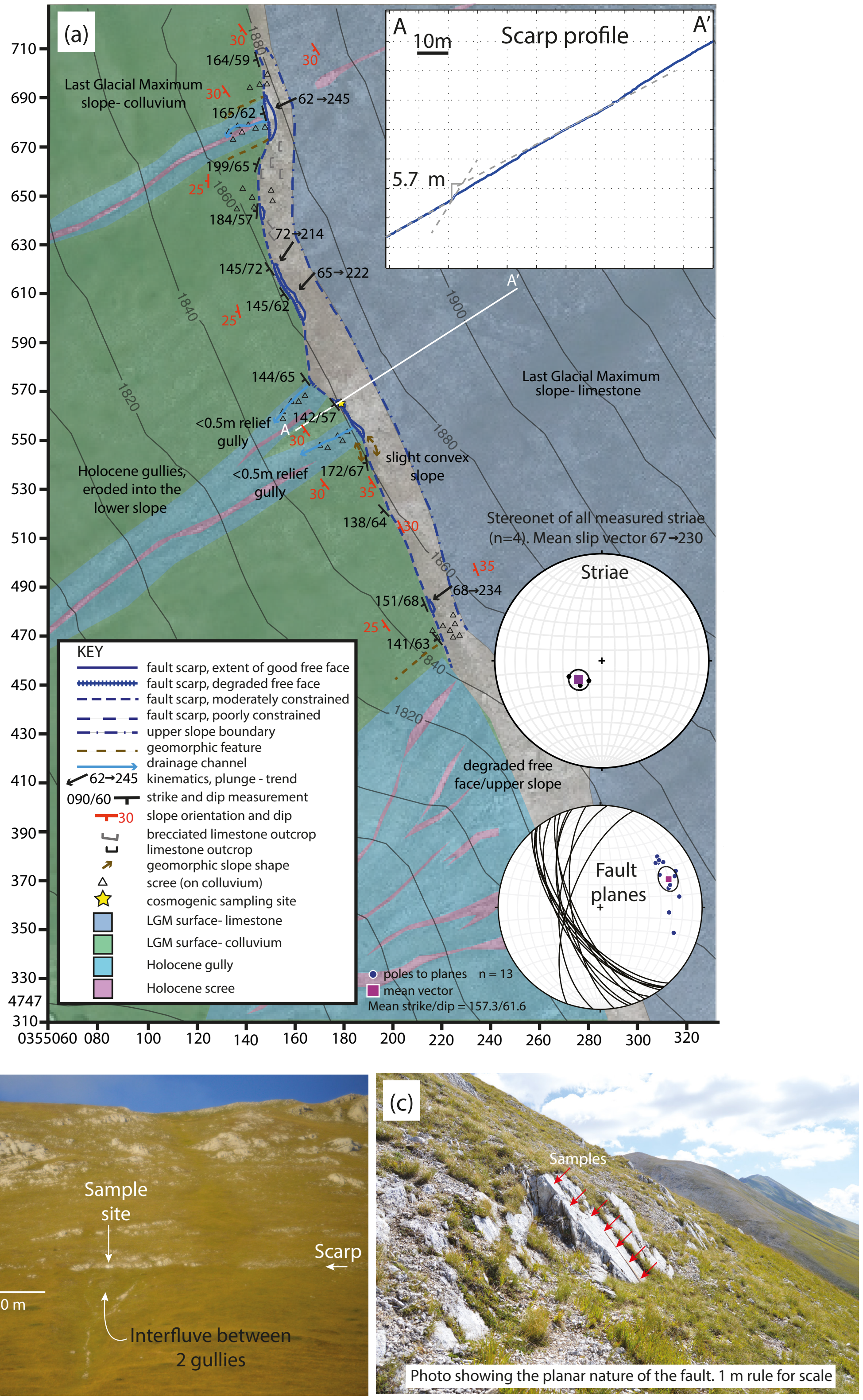

Supplementary Figure 6 - Site characterisation for ^{36}Cl sample site on the Mt. Vettore fault. a) Structural and geomorphic map of the area (overlayed on Google Earth imagery) around the sample site (previously published in ref.[2]), b) View onto the sample site with the key geomorphic features annotated, c) View onto the sample site showing the planar nature of the fault plane and locations of samples. All field photos were taken and annotated by the authors.

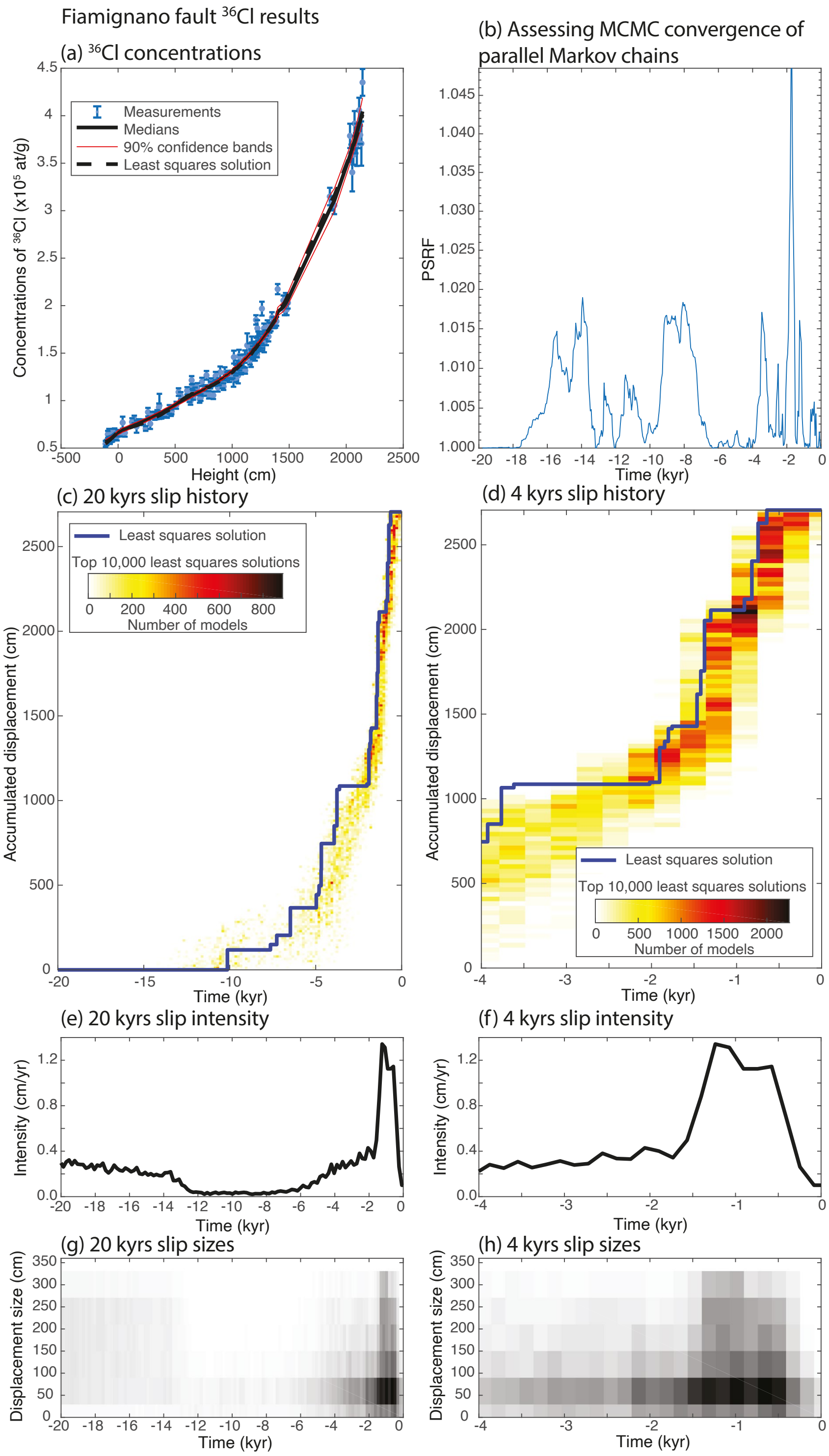

Supplementary Figure 7 – ^{36}Cl concentrations and associated slip history inversion for the Fiamignano fault. a) Measured ^{36}Cl concentrations and modelled concentrations, b) Values for Potential Scale Reduction Factor (PSRF), PSRF < 1.05 indicates MCMC convergence between parallel Markov chains, c) 20 kyrs slip history with ensembles of least squares solutions, d) 4 kyrs slip history with ensembles of least squares solutions, e) 20 kyrs slip intensity, f) 4 kyrs slip intensity, g) 20kyrs slip sizes, h) 4 kyrs slip sizes. Panels a, e, f, g and h show the ensembles of highest likelihood solutions that take account of uncertainties and are penalized by priors. Panels g and h show events from posterior samples (with grey scale to indicate frequencies).

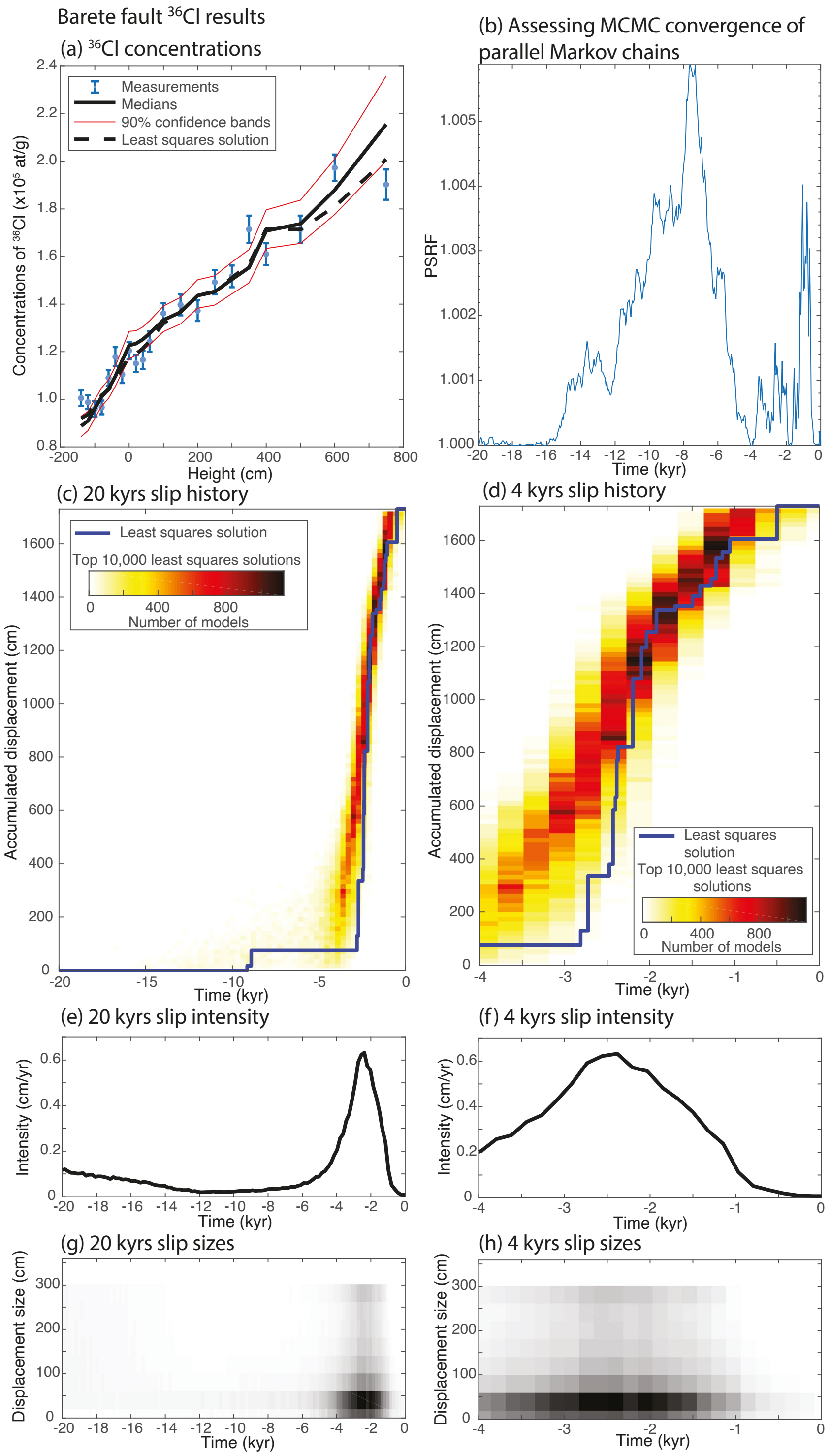

Supplementary Figure 8 – ^{36}Cl concentrations and associated slip history inversion for the Barete fault. a) Measured ^{36}Cl concentrations and modelled concentrations, b) Values for Potential Scale Reduction Factor (PSRF), PSRF < 1.05 indicates MCMC convergence between parallel Markov chains, c) 20 kyrs slip history with ensembles of least squares solutions, d) 4 kyrs slip history with ensembles of least squares solutions, e) 20 kyrs slip intensity, f) 4 kyrs slip intensity, g) 20kyrs slip sizes, h) 4 kyrs slip sizes. Panels a, e, f, g and h show the ensembles of highest likelihood solutions that take account of uncertainties and are penalized by priors. Panels g and h show events from posterior samples (with grey scale to indicate frequencies).

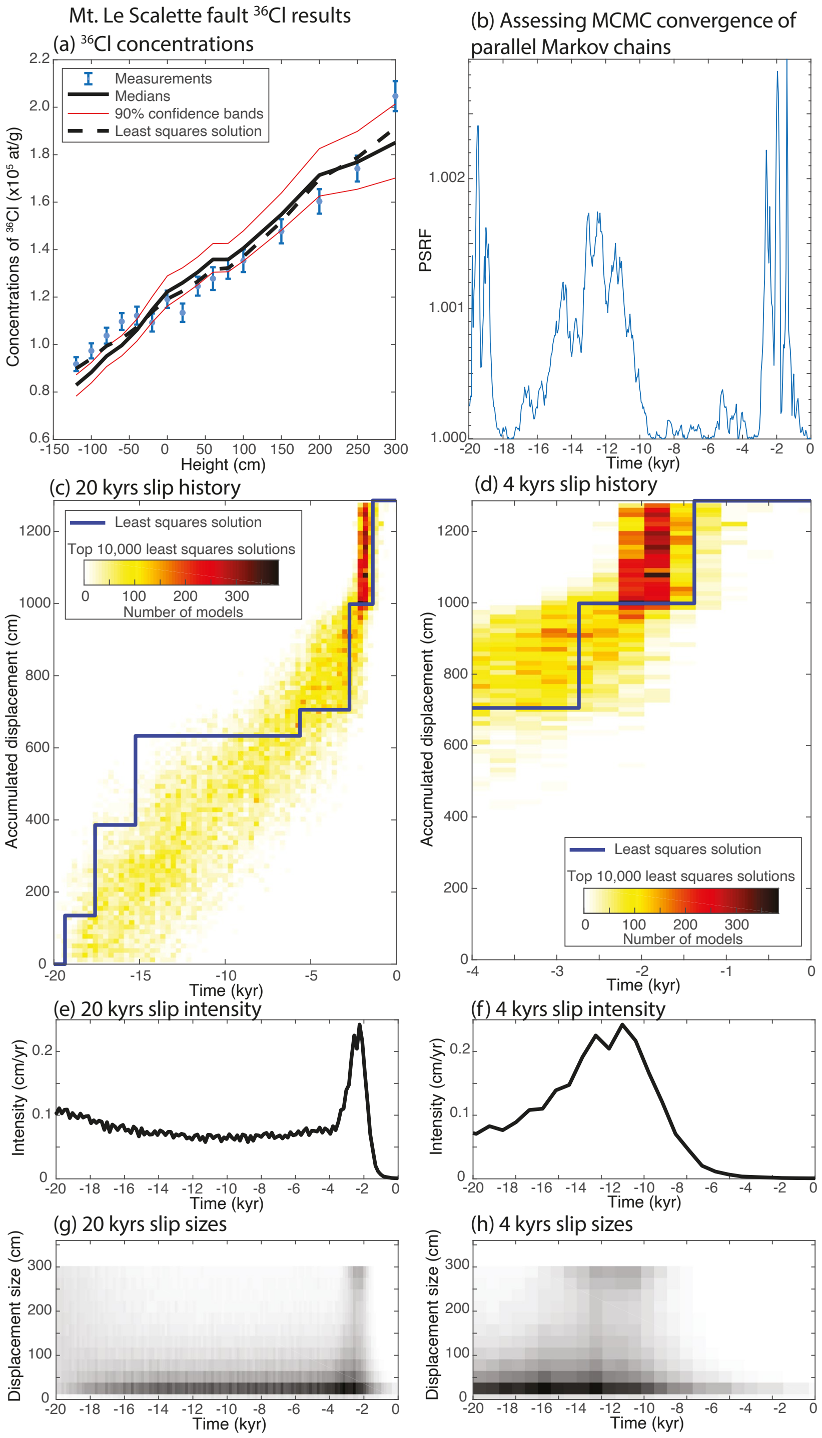

Supplementary Figure 9 – ^{36}Cl concentrations and associated slip history inversion for the Mt. Le Scalette fault. a) Measured ^{36}Cl concentrations and modelled concentrations, b) Values for Potential Scale Reduction Factor (PSRF), PSRF < 1.05 indicates MCMC convergence between parallel Markov chains, c) 20 kyrs slip history with ensembles of least squares solutions, d) 4 kyrs slip history with ensembles of least squares solutions, e) 20 kyrs slip intensity, f) 4 kyrs slip intensity, g) 20kyrs slip sizes, h) 4 kyrs slip sizes. Panels a, e, f, g and h show the ensembles of highest likelihood solutions that take account of uncertainties and are penalized by priors. Panels g and h show events from posterior samples (with grey scale to indicate frequencies).

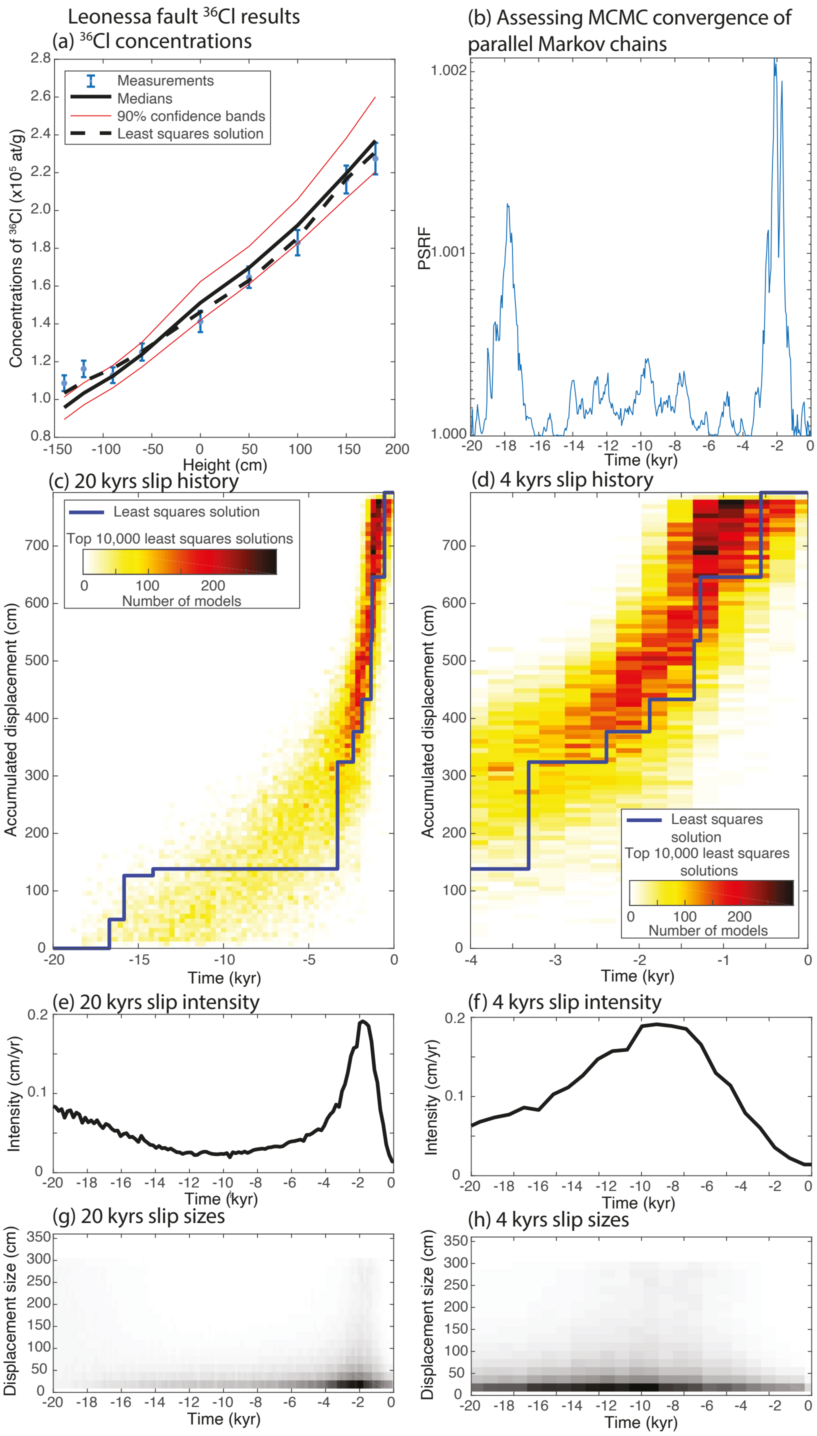

Supplementary Figure 10 – ^{36}Cl concentrations and associated slip history inversion for the Leonessa fault. a) Measured ^{36}Cl concentrations and modelled concentrations, b) Values for Potential Scale Reduction Factor (PSRF), PSRF < 1.05 indicates MCMC convergence between parallel Markov chains, c) 20 kyrs slip history with ensembles of least squares solutions, d) 4 kyrs slip history with ensembles of least squares solutions, e) 20 kyrs slip intensity, f) 4 kyrs slip intensity, g) 20kyrs slip sizes, h) 4 kyrs slip sizes. Panels a, e, f, g and h show the ensembles of highest likelihood solutions that take account of uncertainties and are penalized by priors. Panels g and h show events from posterior samples (with grey scale to indicate frequencies).

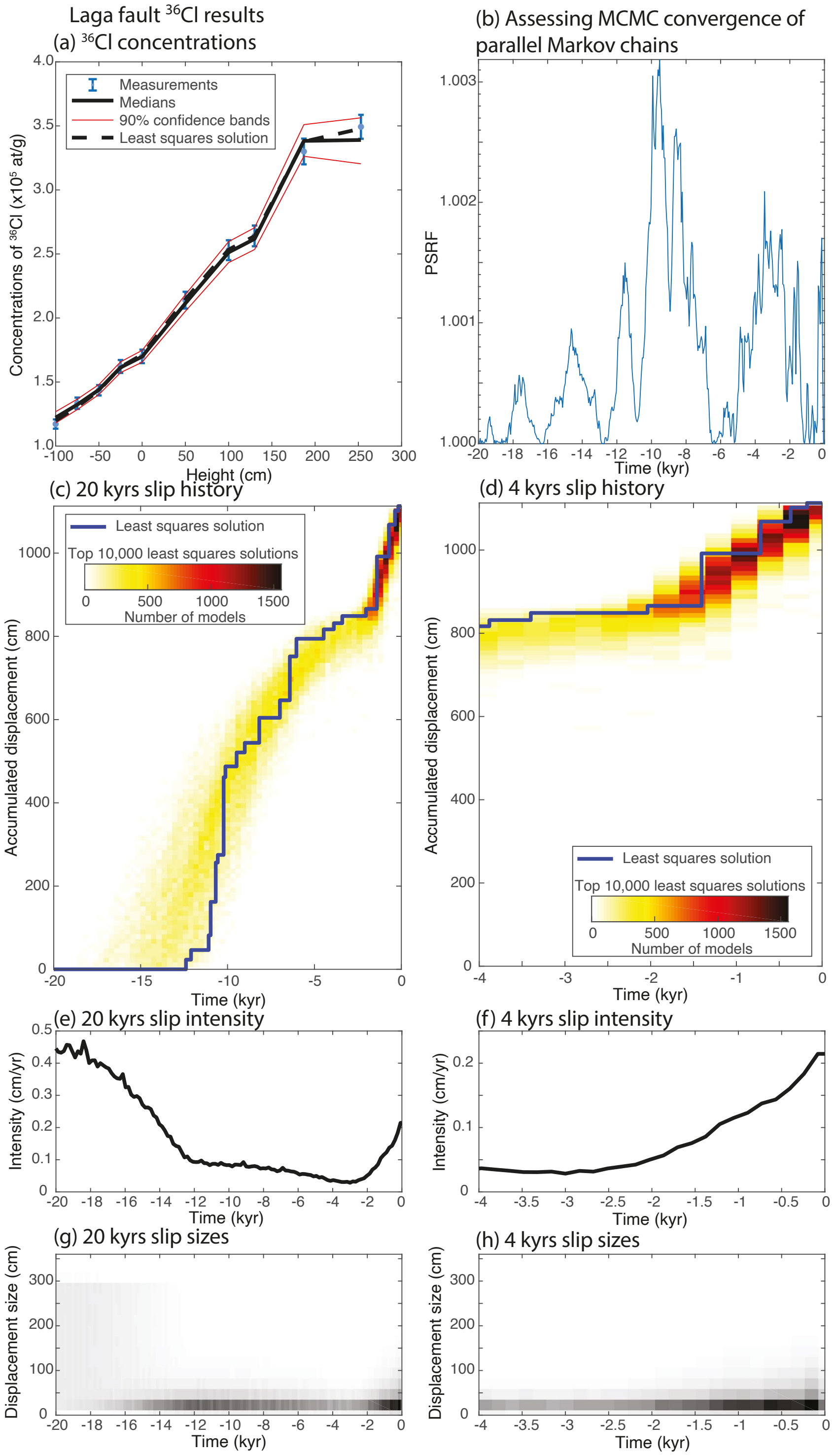

Supplementary Figure 11 – ^{36}Cl concentrations and associated slip history inversion for the Laga fault. a) Measured ^{36}Cl concentrations and modelled concentrations, b) Values for Potential Scale Reduction Factor (PSRF), PSRF < 1.05 indicates MCMC convergence between parallel Markov chains, c) 20 kyrs slip history with ensembles of least squares solutions, d) 4 kyrs slip history with ensembles of least squares solutions, e) 20 kyrs slip intensity, f) 4 kyrs slip intensity, g) 20kyrs slip sizes, h) 4 kyrs slip sizes. Panels a, e, f, g and h show the ensembles of highest likelihood solutions that take account of uncertainties and are penalized by priors. Panels g and h show events from posterior samples (with grey scale to indicate frequencies).

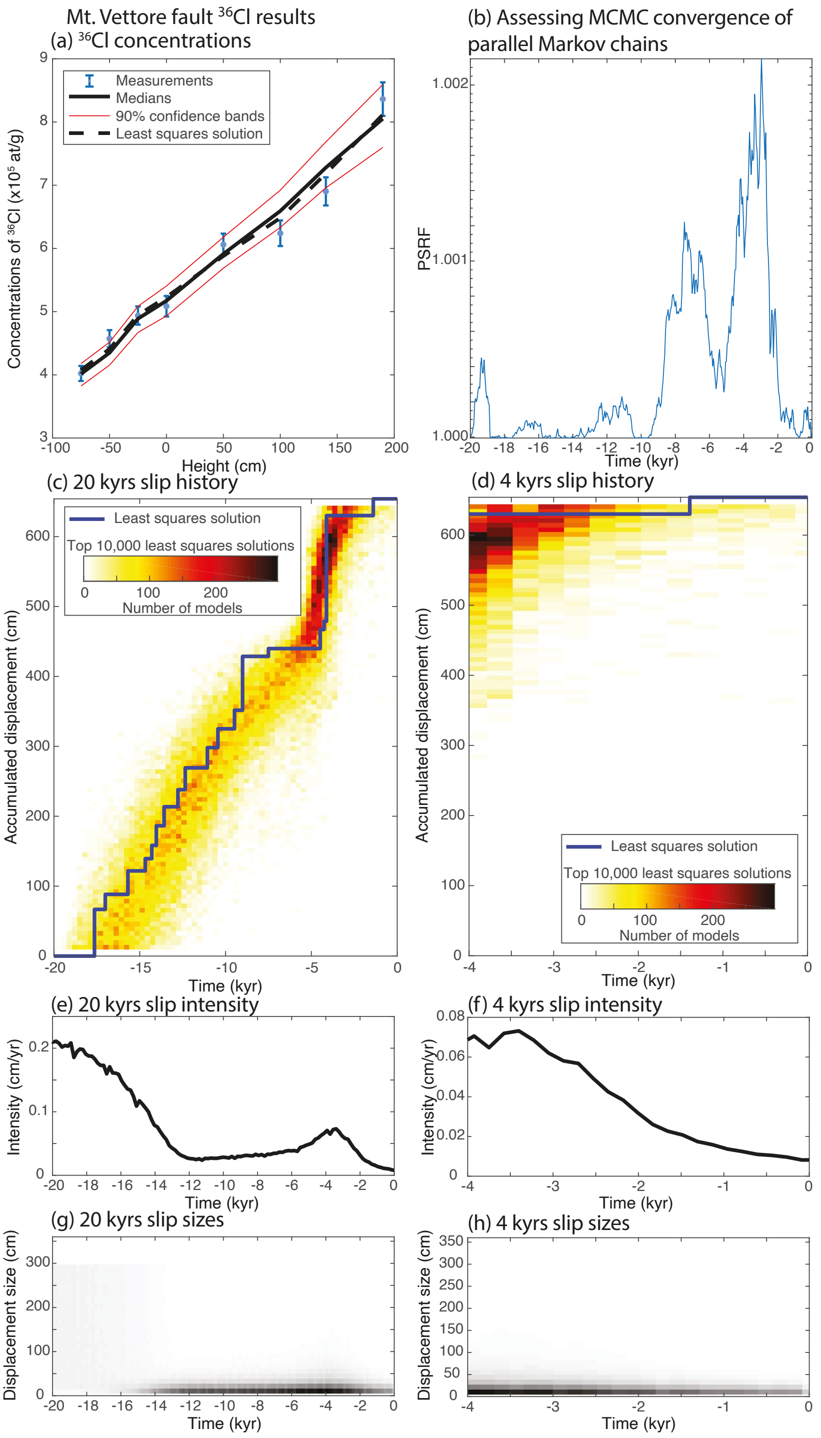

Supplementary Figure 12 – ^{36}Cl concentrations and associated slip history inversion for the Mt. Vettore fault. a) Measured ^{36}Cl concentrations and modelled concentrations, b) Values for Potential Scale Reduction Factor (PSRF), PSRF < 1.05 indicates MCMC convergence between parallel Markov chains, c) 20 kyrs slip history with ensembles of least squares solutions, d) 4 kyrs slip history with ensembles of least squares solutions, e) 20 kyrs slip intensity, f) 4 kyrs slip intensity, g) 20kyrs slip sizes, h) 4 kyrs slip sizes. Panels a, e, f, g and h show the ensembles of highest likelihood solutions that take account of uncertainties and are penalized by priors. Panels g and h show events from posterior samples (with grey scale to indicate frequencies).

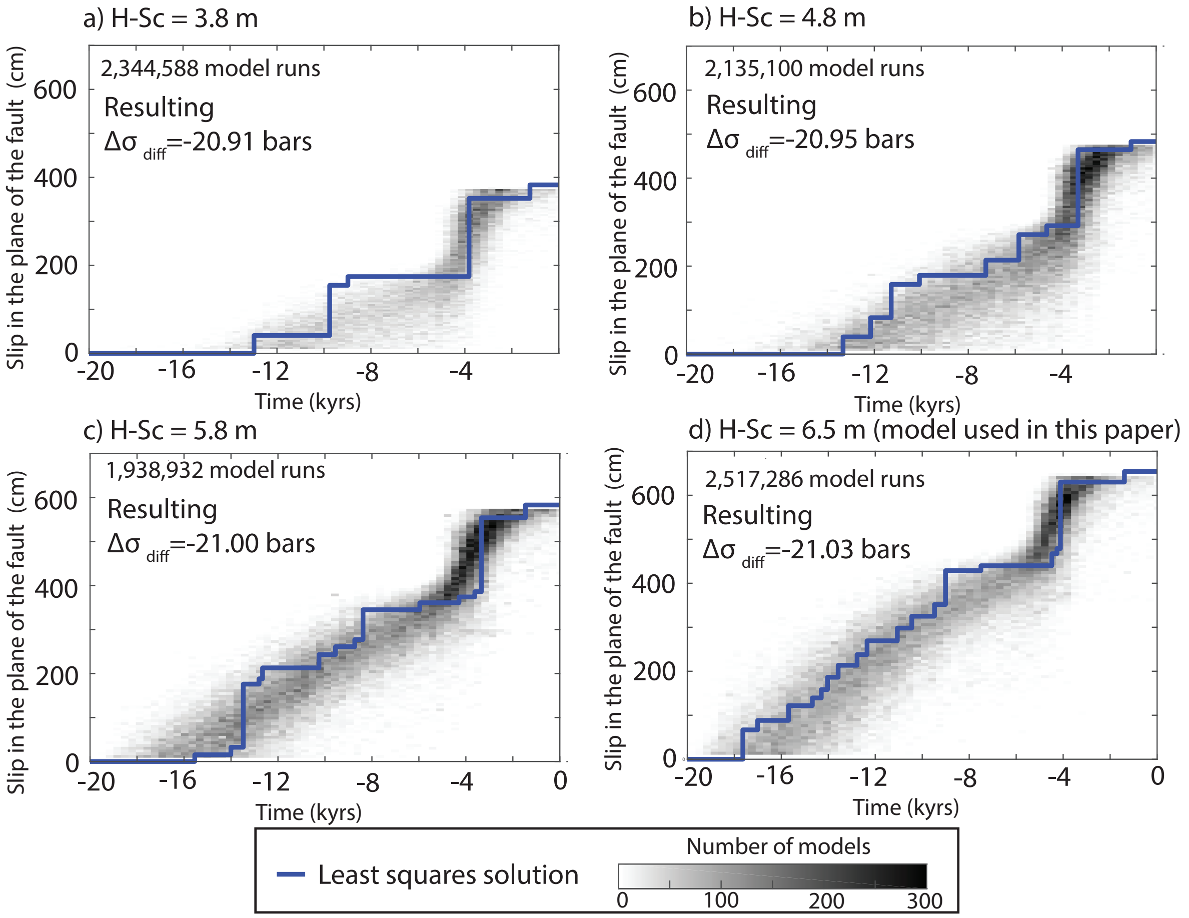

Supplementary Figure 13 – Effect of uncertainty in H-Sc (total slip in the plane of the fault) on the MCMC inverted slip history and resultant differential stress change ($\Delta\sigma_{diff}$) for the Mt. Vettore fault scarp. This figure shows that the derived slip-histories are relatively insensitive to metre-scale errors in throw across the scarps that may occur due to scarp profile construction and interpretation. Repeat surveys provide confidence that errors in throw across the scarps are, in general, <± 1m and therefore we are confident in the first-order patterns of the slip history derived from our modelling.

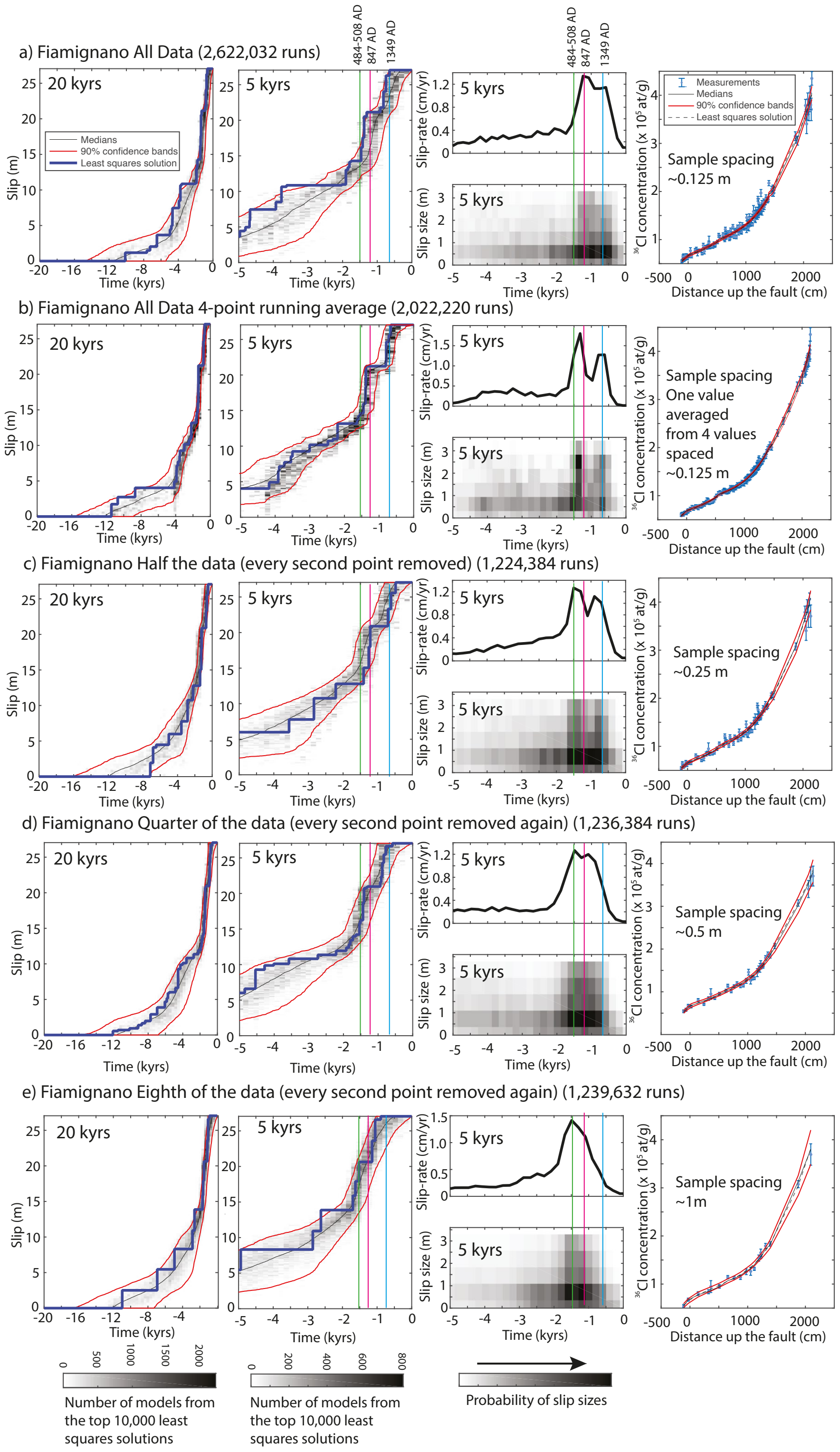

Supplementary Figure 14 - Degradation of Fiamignano data to investigate sensitivity to sample spacing. We progressively degrade the dense sampling for the Fiamignano fault to investigate the effect of lower sampling resolution on other fault scarps. The data is degraded to a point where two well-constrained historical earthquake sequences (1349 AD; 485-505 to 847AD, see ref.[1]) resolvable with the full data disappear. This supplement shows that the <~0.5 m ^{36}Cl sample spacing we have achieved for the sites analysed is adequate to resolve the slip-rate changes we claim, and thus lends confidence to our interpretation of other sites in the paper.

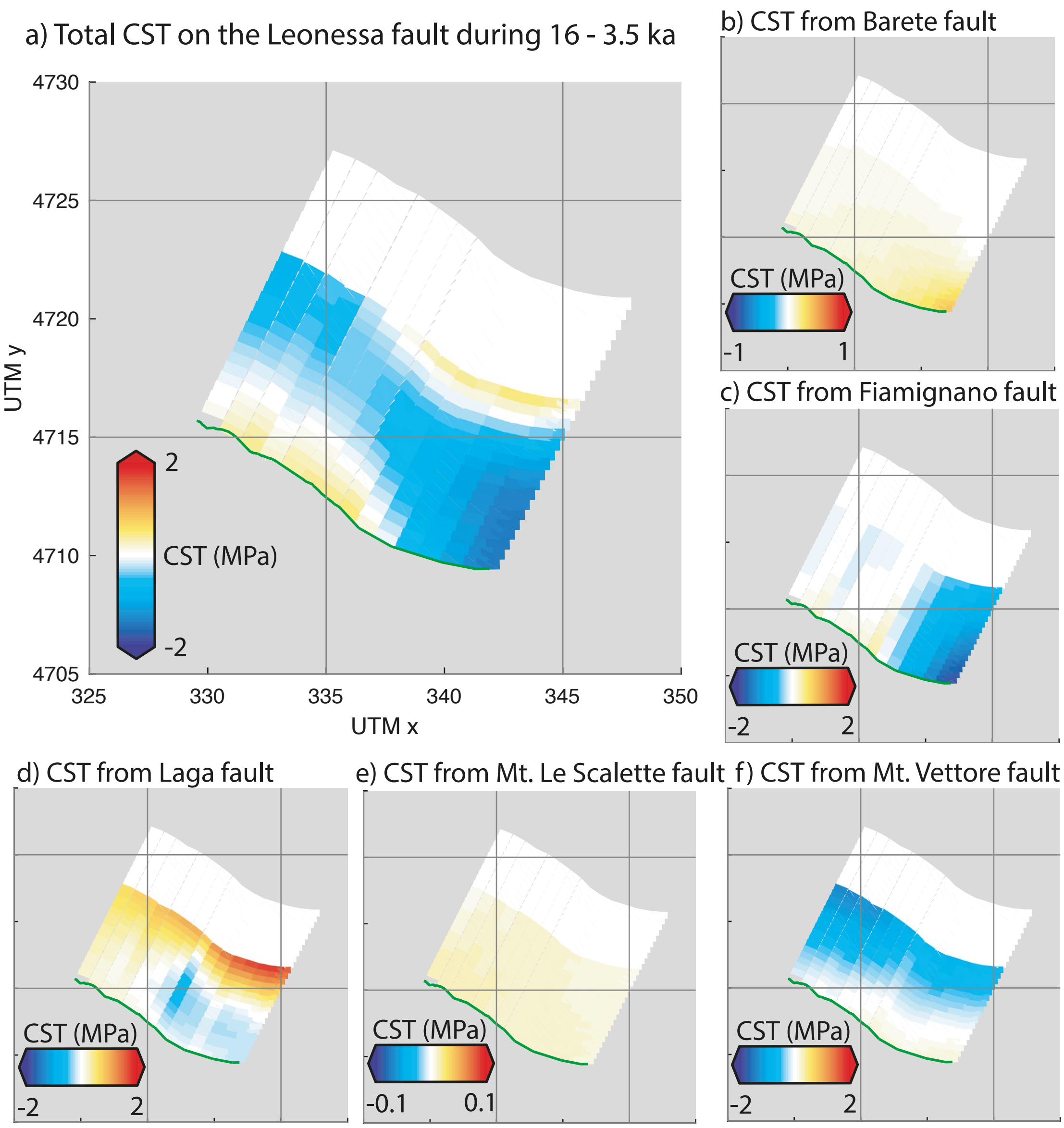

Supplementary Figure 15 – Total Coulomb Stress Transferred (CST) on the Leonessa fault from contributing faults during the period of quiescence from 16 – 3.5 ka (as evidenced from ^{36}Cl analyses). a) Total CST accumulated on the Leonessa fault, b) CST contribution from slip on the Barete fault, c) CST contribution from slip on the Fiamignano fault, d) CST contribution from slip on the Laga fault, e) CST contribution from slip on the Mt. Le Scalette fault, f) CST contribution from slip on the Mt. Vettore fault.

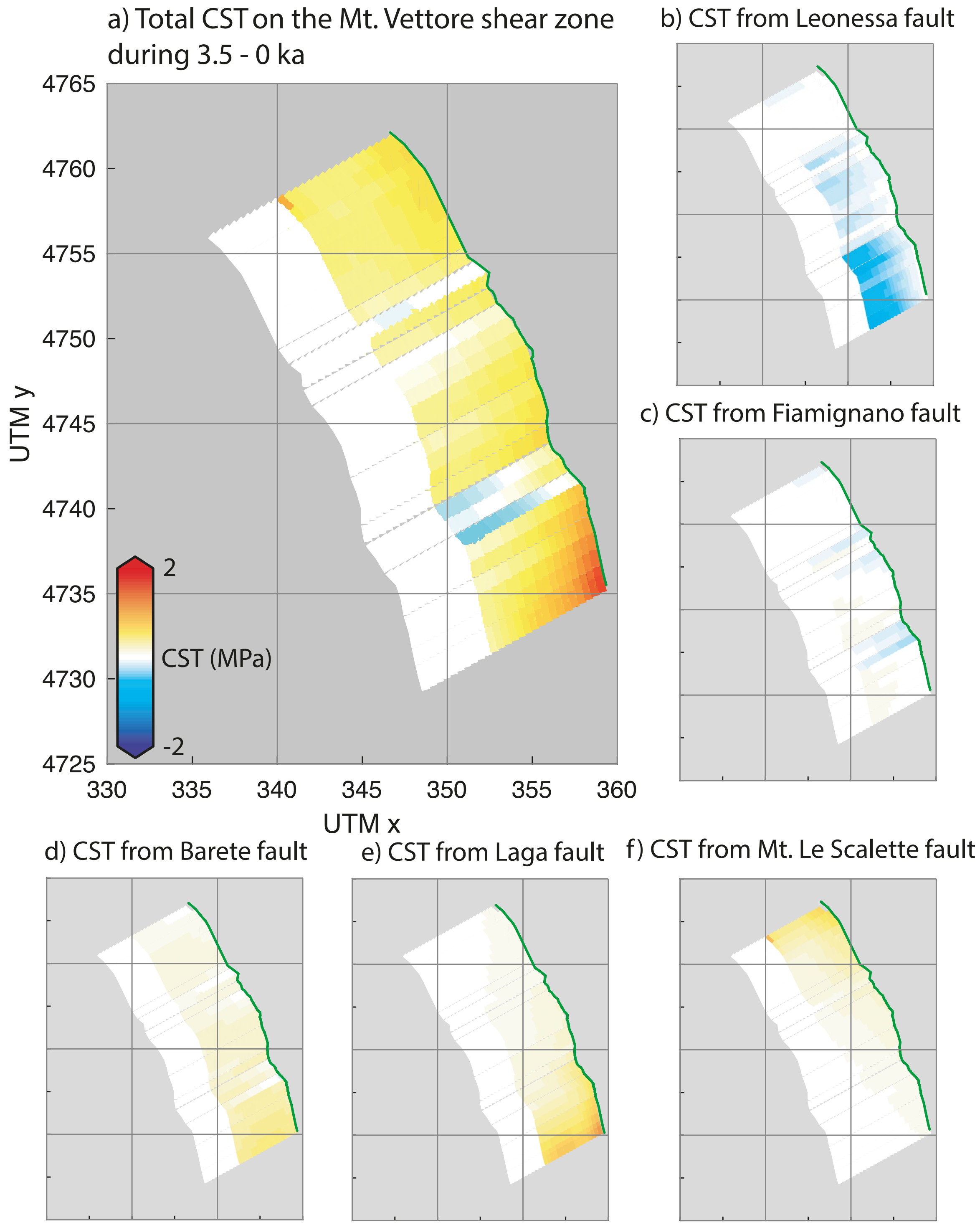

Supplementary Figure 16 – Total Coulomb Stress Transferred (CST) on the Mt. Vettore fault from contributing faults during the period of quiescence from 3.5 - 0 ka (as evidenced from ^{36}Cl analyses). a) Total CST accumulated on the Mt. Vettore fault, b) CST contribution from slip on the Leonessa fault, c) CST contribution from slip on the Fiamignano fault, d) CST contribution from slip on the Barete fault, e) CST contribution from slip on the Laga fault, f) CST contribution from slip on the Mt. Le Scalette fault.

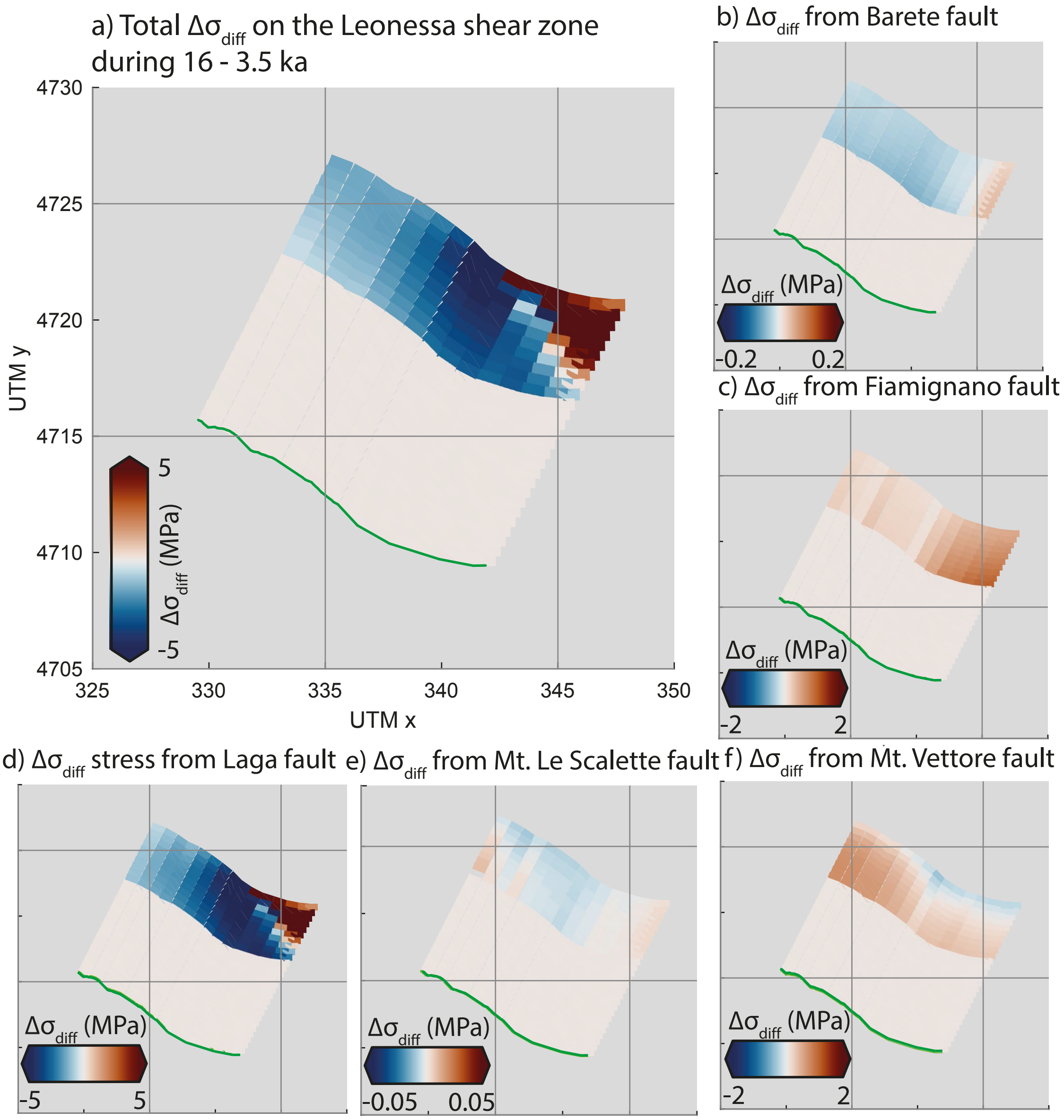

Supplementary Figure 17 – Total differential stress ($\Delta\sigma_{diff}$) on the Leonessa shear zone from contributing faults during the period of quiescence from 16 – 3.5 ka (as evidenced from ^{36}Cl analyses). a) Total $\Delta\sigma_{diff}$ accumulated on the Leonessa fault, b) $\Delta\sigma_{diff}$ contribution from slip on the Barete fault, c) $\Delta\sigma_{diff}$ contribution from slip on the Fiamignano fault, d) CST contribution from slip on the Laga fault, e) $\Delta\sigma_{diff}$ contribution from slip on the Mt. Le Scalette fault, f) $\Delta\sigma_{diff}$ contribution from slip on the Mt. Vettore fault.

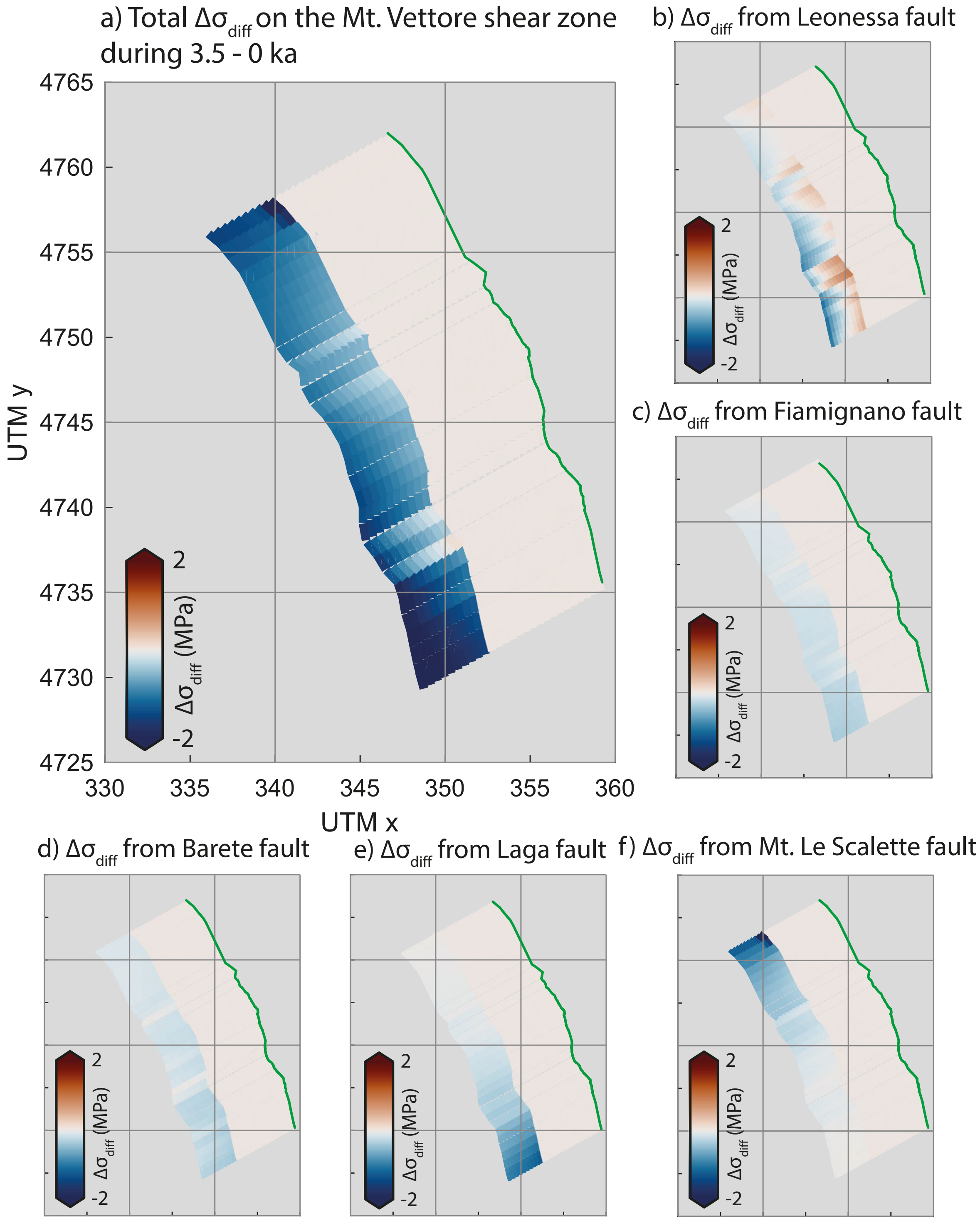

Supplementary Figure 18 – Total differential stress ($\Delta\sigma_{diff}$) on the Mt. Vettore shear zone from contributing faults during the period of quiescence from 3.5 - 0 ka (as evidenced from ^{36}Cl analyses). a) Total $\Delta\sigma_{diff}$ accumulated on the Mt. Vettore fault, b) $\Delta\sigma_{diff}$ contribution from slip on the Leonessa fault, c) $\Delta\sigma_{diff}$ contribution from slip on the Fiamignano fault, d) $\Delta\sigma_{diff}$ contribution from slip on the Barete fault, e) $\Delta\sigma_{diff}$ contribution from slip on the Laga fault, f) $\Delta\sigma_{diff}$ contribution from slip on the Mt. Le Scalette fault.

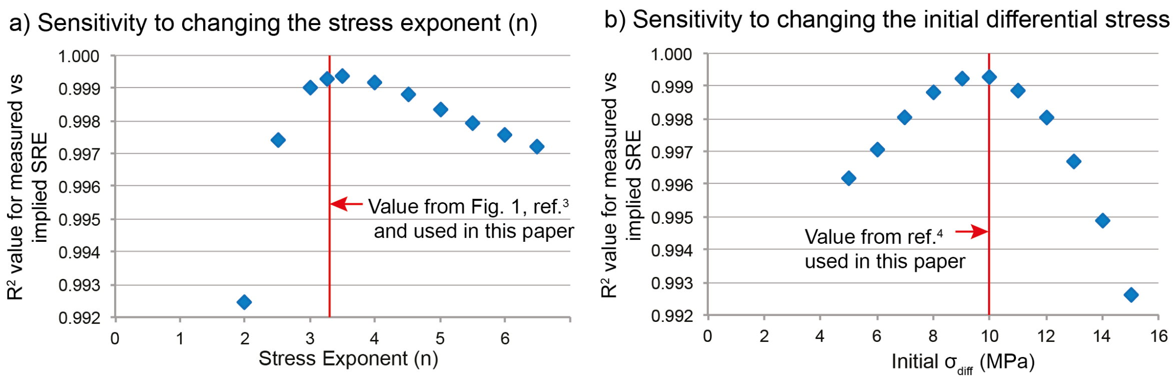

Supplementary Figure 19 - Sensitivity tests for chosen values of stress exponent[3] and absolute differential stress[4] (σ_{diff}) at 15-24 km. Our chosen values from the literature show a high R^2 value (i.e. low misfit) between measured and implied Slip Rate Enhancement (SRE).

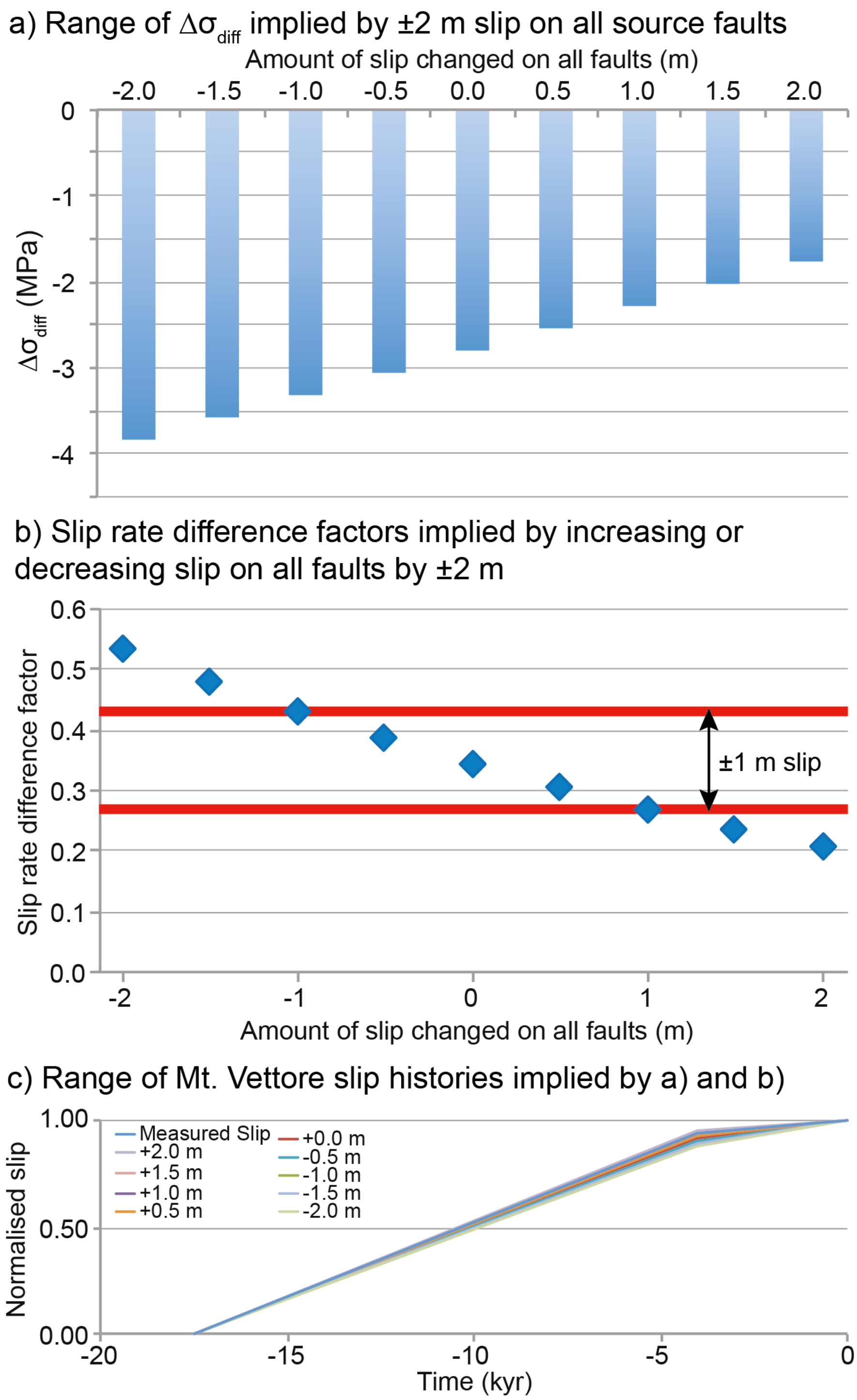

Supplementary Figure 20 - Sensitivity test of a) the calculated differential stresses, b) the slip rate difference factor which compares the slip rate in clusters/anti-clusters, and c) implied slip-rate histories associated with the interpreted values of slip across the fault scarps. We calculate the effect of assuming that throw values for all scarps could be ±2 m (a range bigger than our estimate of the uncertainty for individual scarps made in the field which is in the range of ±1 m, shown by the red lines). The similarity between implied slip histories in (c) suggests our overall conclusions are relatively insensitive to the interpreted slip across the scarps.

Fault (Site ID)	Elevation (m)	Latitude (˚N)	Longitude (˚E)	α[1] (˚)	β[2] (˚)	γ[3] (˚)	Scarp H-Sc[4] (cm)	Trench H-tr[4] (cm)
Leonessa (L)	1063	42.56138	12.95964	25.5	63.0	26.7	793	140
Barete (BAR)	871	42.46875	13.26943	18.0	40.0	31.0	1730	140
Mt. Vettore (MV)	1800	42.86695	13.22707	29.0	60.0	29.0	654	75
Mt. Le Scalette (CO)	975	43.06345	12.91527	14.0	60.0	29.0	1286	120
Laga (LA)	1502	42.59829	13.36777	29.0	54.0	29.0	1112.5	100

(1) Dip of the colluvial wedge

(2) Dip of the preserved bedrock fault scarp

(3) Dip of the upper slope

(4) Calculated in the plane of the fault

Supplementary Table 1 – Scarp modelling parameters used for ^{36}Cl modelling. Parameters used for modelling the ^{36}Cl samples from the Fiamignano fault are published in ref. [1]. The Site ID given corresponds to the site IDs used in Supplementary Data 1.

Fault name	Fault length (km)	Fault dip (•)	Downdip length (km)	Fault area (km²)	M_{max}	ASS/MS	ASS/AS	Max. slip (m)	Slip @ cosmo site (m)
Barete	19.7	42	22.4	441.6	6.66	0.71	1.41	2.40	0.64
Fiamignano	30.7	53	18.8	576.6	6.78	0.70	1.39	3.10	1.22
Laga	30.2	53	18.8	567.2	6.77	0.72	1.39	3.00	1.16
Leonessa	14.3	62	17.0	242.9	6.41	0.69	1.38	2.00	0.43
Mt. Le Scalette	18.0	62	17.0	305.8	6.51	0.68	1.40	2.40	0.83
Mt. Vettore	32.9	63	17.0	558.9	6.76	0.69	1.32	3.20	1.13

Supplementary Table 2 – Parameters used to calculate the characteristic earthquake magnitude modelled on the faults discussed and to constrain the proportion of slip that occurs at the surface compared to depth. The concentric slip distribution assumes a symmetrical triangular surface slip distribution. ASS/MS = Average SubSurface displacement/Mean Surface displacement. AS/MS = Average subsurface displacement/Average Surface displacement.

Supplementary References

1. Cowie, P. A. *et al.* Orogen-scale uplift drives episodic behaviour of earthquake faults. *Nat. Sci. Rep.* 1–10 (2017) doi:10.1038/srep44858.
2. Mildon, Z. K. The link between earthquakes and structural geology; the role of elapsed time, 3D geometry and stress transfer in the central Apennines, Italy. (University College London, 2017).
3. Cowie, P. A., Scholz, C. H., Roberts, G. P., Faure Walker, J. P. & Steer, P. Viscous roots of active seismogenic faults revealed by geologic slip rate variations. *Nat Geosci* **6**, 1036–1040 (2013).
4. Behr, W. M. & Platt, J. P. A naturally constrained stress profile through the middle crust in an extensional terrane. *Earth Planet Sci Lett* **303**, 181–192 (2011).
