# Supplementary material for: Surface faulting earthquake clustering controlled by fault and shear-zone interactions
Source: Nat Commun. 2022 Nov 21;13:7126. doi: 10.1038/s41467-022-34821-5 (PMC9681762; doi:10.1038/s41467-022-34821-5)
Supplement: Supplementary file 2 — Description of Additional Supplementary files [file 41467_2022_34821_MOESM2_ESM.pdf]

Description of Additional Supplementary Files

Filename: Supplementary Data 1
Description: Spreadsheet with 36Cl and elemental composition data for five of the faults analysed. Data for the Fiamignano fault are already published and can be found in Cowie et al. (2017). AMS data are available from the NERC BGS data repository from https://www.bgs.ac.uk/services/ngdc/accessions/index.html#item128345.

Filename: Supplementary Data 2
Description: Spreadsheet with four tabs used to calculate the total CST for 1.) Leonessa and 2.) Mt. Vettore brittle faults during periods of quiescence, and the total differential stress for 3.) Leonessa and 4. Mt. Vettore shear zones during the same time periods.
The differential stress is calculated by using sig_reverse = τ and dip – 90 = β in the equation $\tau = \frac{1}{2}(\sigma_1 - \sigma_3)sin2\beta$ (King et al. (1994)). The minimum value of differential stress at the top of the shear zone is also extracted – this is the rate-limiting element and this value is used in Supplementary Data 3 to calculate the change in strain rate. Minimum values of differential stress for different depths are also extracted and used in Supplementary Data 3.

Filename: Supplementary Data 3
Description: Spreadsheet which details how we derived strain-rate from values of change in differential stress, and how we converted these into implied slip-rate for those measured using 36Cl, following the step-by-step approach laid out in Figure 3. The first two tabs are calculating the strain rate, earthquake recurrence and slip histories for Mt Vettore and Leonessa faults. The “Combined” tab brings the comparison of slip histories together (which is included in Figure 6). The “Depth variation” tab demonstrates that the strain rate can be calculated for different depths.
